# The use of a candidate gene approach to study *Botrytis cinerea* resistance in *Gerbera hybrida*

**DOI:** 10.3389/fpls.2023.1100416

**Published:** 2023-03-22

**Authors:** Yiqian Fu, Yin Song, Jaap M. van Tuyl, Richard G. F. Visser, Paul Arens

**Affiliations:** ^1^ Plant Breeding, Wageningen University and Research, Wageningen, Netherlands; ^2^ Phytopathology, Wageningen University and Research, Wageningen, Netherlands

**Keywords:** HRM, QTL haplotypes, MAS, gene localization, functional allele, TRV, susceptibility genes

## Abstract

Candidate genes (CG) for *Botrytis cinerea* resistance described in literature were mapped on gerbera linkage maps for which several QTL for Botrytis resistance had been found previously using a rapid, low-cost platform for SNP genotyping. In total, 29 CGs were mapped in either of two mapping populations. Four CGs were mapped within the previous identified QTL intervals and three co-localized with QTL. Two of these CGs for resistance against *B. cinerea*, *PG1* (polygalacturonase gene) and *sit* (*sitiens*, ABA-aldehyde oxidase gene) that mapped in QTL regions for the ray floret disease resistance test were studied in detail. Virus-induced gene silencing (VIGS) was used for gene function analysis to determine the CGs’ role in gerbera resistance to Botrytis. Ray florets, of which the CGs were silenced, showed a significantly delayed growth of lesions upon Botrytis infection compared to controls. Combining QTL analysis, candidate gene mapping and VIGS showed to be an useful combination to identify possible causal genes and for understanding the molecular mechanisms of Botrytis resistance in gerbera. The two genes seem to act as partial S-genes and are likely among the determining genes leading to the variation observed for *B. cinerea* resistance in gerbera.

## Introduction

Gerbera is an economically important ornamental plant, which is mainly used as cut flower. In pre-harvest and post-harvest processes, high relative humidity regularly occurs, which can lead to grey mold infections and subsequent major losses in gerbera production. For instance, economical loss was estimated at € 2.5 million in 2007 in The Netherlands alone (Marcelis, unpublished). Gerbera grey mold may be suppressed by management practices and fungicide application during production in greenhouses, yet prevention and control of this disease during transportation or after sales is difficult.

Traditionally, breeding in gerbera is performed by F1 crossing and (recurrent) selection in a number of generations. Although this traditional method is considered time and labor consuming, it is still widely in use for gerbera and ornamental plant breeding in general since lots of horticultural traits (e.g. flower color, flower pattern and shape), as well as agronomical traits (e.g. multiplication rate and flower production) are segregating due to the high variation in parental genotypes of these outcrossing crops (Arens et al., 2012; Smulders et al., 2012). These traits can also be easily selected as they are either visible traits (color, pattern, shape) or easily quantifiable traits (multiplication rate and flower production). However, selection for genetic and phenotypic complex quantitative traits like disease resistances, which are often also influenced by environment and present themselves in continuous distributions (Paterson et al., 1988), is very difficult using only visual selection schemes.

Resistance to *B. cinerea*, the cause of gerbera grey mold, is such a quantitative trait where the contributions of multiple loci are needed to reduce disease severity (Mengiste et al., 2003; Poland et al., 2009). In our previous study, 20 QTLs for Botrytis resistance in ray floret, whole and bottom of inflorescence trait were detected in two gerbera mapping populations (Fu et al., 2017). Genetic linkage between a marker and a QTL allele of the trait of interest, can be broken by recombination (Andersen and Lübberstedt, 2003). Furthermore, in cross-pollinating highly diverse ornamental species, there is often an ascertainment bias (Kumar et al., 2000). Observed linked marker alleles which were detected in single F1 outbred populations might not be directly applicable to predict the association with the preferred QTL allele in another population (Williams, 1997; Liu et al., 2011). For ornamental crops, with increasing EST sequence data availability the use of a candidate gene (CG) approach (Debener, 2009; Smulders et al., 2011; Arens et al., 2012) might improve the accuracy of Marker Assisted Selection (MAS) and circumpass the mentioned problems. Developing molecular markers which are derived directly from polymorphic loci in functional CGs will be in complete linkage (Andersen and Lübberstedt, 2003). Through co-localization of CGs with QTLs, promising genes underlying the QTLs could be quickly pinpointed (Pflieger et al., 2001; Decroocq et al., 2005; Norelli et al., 2009; Kawamura et al., 2011; Gardner et al., 2016) and used for understanding molecular mechanisms for traits of interest (Smulders et al., 2011). Like many other ornamental plants, gerbera has a heterozygous genetic background and a lack of adequate genetic information. Instead of going through the process of high-resolution fine mapping and identifying tightly-linked markers for MAS, we developed a CG approach searching for possible causal genes for Botrytis resistance in gerbera.

The infection process of the necrotrophic pathogen, *B. cinerea*, is described by the three stages: penetration of host epidermal cell surface, primary lesion formation, lesion expansion/tissue maceration and sporulation (Jarvis, 1962; van Kan, 2006; Choquer et al., 2007; Valero-Jiménez et al., 2019). During the infection, a series of plant genes, such as genes in cell wall biosynthesis or affecting cell wall composition, in signaling pathways, and in the complex pathways of plant natural product biosynthesis, can be involved in resistance to the pathogen.

Using literature on genes involved in Botrytis resistance and the availability of transcriptome data (Fu et al., 2016) from which a random set of SNP markers was previously used to perform a QTL study for Botrytis resistance (Fu et al., 2017), we now specifically targeted genes known from other species to test a CG approach for Botrytis resistance is an functional option. The first step was to map SNPs found in CGs from literature to look for co-localization of the CG gene positions with previously found QTL positions. In order to validate the approach we also used virus induced gene silencing (VIGS) for two genes to see if silencing of genes found to co-localize with QTL for Botrytis resistance would influence Botrytis disease severity. VIGS systems (Baulcombe, 1999; Becker & Lange, 2010) have been successfully applied in a large number of ornamental plants and as such considered an attractive approach for gene characterization in ornamentals (Jiang et al., 2011), particularly those not amenable to tissue culture or genetic transformation.

## Materials and methods

### Plant materials and Botrytis disease test data

Plant materials were composed of the parents and two gerbera populations previously used for QTL mapping (Fu et al., 2017). In brief, population S containing 276 offspring, was obtained from a cross between breeding lines SP1 and SP2 whereas population F consisting of 270 progeny was derived from a cross between FP1 and FP2. Genomic DNA of the two populations and the four parents were isolated following the DNA isolation protocol of Fulton et al. (1995) with some adaptations. Phenotypic data available for these two F1 progenies were based on three tests of whole inflorescence, bottom (of disc floret) and ray floret, respectively with scores ranging from 0 (no symptom) to 5 (very serious) as described by Fu et al. (2017). All tests were performed with *B. cinerea* (strain B05.10) which was kindly provided by J. van Kan (Wageningen University). In brief, for the Botrytis disease test on whole inflorescence and bottom, a spore suspension of 1 × 10^5^/ml in water was sprayed on the inflorescence with a fine plant sprayer and incubated for 5 days in a climate cell at 20°C and a R.H. of 90%. Whole inflorescences were visually evaluated to score, after which, the bottom of the capitulum was cut (horizontal cross section) to check (score) fungal growth inside the capitulum for the bottom test. The response to Botrytis infection on whole inflorescence and bottom was scored ranging from 0 (no symptom) to 5 (completely rotten). For the ray floret test, inoculation was performed by pipetting 2 μl of a 3 × 10^5^/ml spore suspension in potato dextrose on the upper surface of ray florets and incubated for 48 h at 20°C and nearly 100% relative humidity. Disease scoring was assessed as follows: 0 no visible symptoms; 1 infection limited in inoculation droplet size; 2 lesion extended twice to four times the droplet size; 3 large lesion area but still smaller than half of the ray floret; 4 lesion area larger than half of the ray floret; and 5 complete necrosis. Botrytis scores varied between parents and populations means. Scores in population S for whole inflorescence, bottom and ray floret were 2.42 ± 0.55 (SP1 = 1.0/SP2 = 2.2), 2.96 ± 0.63 (SP1 = 1.7/SP2 = 2.9) and 2.98 ± 0.79 (SP1 = 1.9/SP2 = 1.8) and in population F 3.64 ± 0.40 (FP1 = 3.4/FP2 = 3.6), 3.80 ± 0.40 (FP1 = 3.4/FP2 = 4.1) and 3.14 ± 0.80 (FP1 = 4.2/FP2 = 2.3), respectively.

### Candidate gene genotyping

Literature was screened for genes involved in Botrytis resistance. Sequences of CGs associated with Botrytis resistance were run against the gerbera ESTs database (Fu et al., 2016) using tBLASTn in the blast-2.2.28+ program. Within the selected best-hit gerbera EST sequence, heterozygous SNPs in just one parent were identified as potential marker and Open Reading Frames (ORFs) were identified to avoid SNPs close to intron/exon boundaries. High Resolution Melting (HRM, LightScanner, Idaho Technology) analysis was used for genotyping. Primer pairs were designed to amplify 80 to 150 bp fragments using Primer3 online (Untergasser et al., 2012) and first tested on the parents and four randomly selected offspring. Only when the melting curve of the two parents and selected individuals could be clearly distinguished, the markers were used for whole population genotyping.

Segregation ratios of the two allelic offspring groups (heterozygous vs homozygous) were tested by χ² (1:1) statistics (95% CI) and the mean of the disease scores between these two groups from all three disease tests was tested for significance (P<0.05) by T-test using SPSS software (Version 21). CGs were mapped on the individual parental linkage maps previously constructed (Fu et al., 2017) by using JoinMap^®^4 (van Ooijen, 2006). QTL analyzes, with CG positions added to the map, were repeated as described earlier (Fu et al., 2017). Because not all individuals from the original mapping population were available, results were slightly different from previous results of Fu et al. (2017) and only used for confirmation.

### Allele identification of candidate genes

Haplotype information of promising CGs in the four parents was obtained using the sequence and SNP positions in their transcriptome data (Fu et al., 2016) and these were supplemented by Sanger sequencing to acquire a longer and if possible full-length gene sequence. Primers for amplifying CG fragments from genomic DNA for Sanger sequencing were designed by Primer3 online. PCR conditions were in Fu et al. (2017). PCR products, showing single bands with expected size, were purified using the QIAquick PCR Purification Kit (Qiagen) and cloned into the pGEM^®^-T Easy Vector System I (Promega). Multiple positive clones were sequenced using Sanger sequencing

### Candidate gene expression analysis after Botrytis inoculation

*B. cinerea* spore suspension (3 × 10^5^/ml) was inoculated on the upper side of ray florets. Infected ray florets were collected at different time points (0 hpi, 6 hpi, 12 hpi, 24 hpi, 36 hpi, 48 hpi and 72 hpi at initial experimental setup) whereas mock samples were taken at 24 hpi only. Single ray floret (for FP2 due to smaller size three ray florets) were put in an Eppendorf with two metal balls frozen in liquid nitrogen and stored at -80 ^0^C upon gene expression analysis.

RNA from infected ray florets was extracted using Trizol (Life Technologies) according to the manufacturer’s protocol with a small modification (Fu et al., 2016). Quality and quantity of RNA samples was checked on 1% agarose gel and by NanoDrop. cDNA synthesis according to the iScript™ cDNA Synthesis Kit (Bio-Rad) was used for Real time quantitative PCR (RT-qPCR). The same primers as used for HRM analysis and reference gene GAPDH obtained from Deng et al. (2014) were used for SYBR green qRT-PCR. Reference and CGs were always run together with three biological replicates and three technical replicates.

Relative quantification method (Livak and Schmittgen, 2001) was used to analyze data. The change in expression of candidate (target) gene was normalized to the reference gene expression and presented as fold change.

### VIGS vector construction

Sequences of CGs *ghPG1* and *ghsit* were retrieved from the gerbera EST database and the supplementary Sanger sequencing whereas reporter genes were retrieved from the gerbera EST database only. Gene-specific primers, added with *att*B1 and *att*B2 adapter, were designed using Primer3 online (listed in **Table S1**). The expected fragments of target genes were divided into 20 bp sequences to blast against our gerbera EST database to check whether they may trigger an off-target gene silencing. The fragments of the CGs (*ghPG1*, *ghsit*) and three reporter genes (*ghPDS*, *ghCHS1*, *ghCHS4*) for VIGS were amplified from gerbera cDNA. Furthermore to co-suppress the two CGs (*ghPG1*+*ghsit*) simultaneously a construct was made consisting of both gene fragments.

The Gateway-compatible Tobacco rattle virus (TRV) two-component Agrobacterium mediated expression system was used for gene silencing as previously described (Liu et al., 2002). DNA fragments of reporter genes and CGs were individually cloned into pDONR207 to generate entry vectors. Entry vectors with the CG fragment insertion, were verified by sequencing using primers *att*L1 and *att*L2 listed in **Table S1**. Target gene fragments were subsequently cloned into the destination vector pTRV2 to generate constructs *TRV2::GOI* (Gene Of Interest). A negative control TRV2 construct (*TRV2::ghGUS*) carrying a 648 bp *GUS* fragment was also used as described by Song and Thomma (2016). All *TRV2* constructs were confirmed by DNA sequencing (using TRV2 primers; **Table S1**).

All *TRV1* and *TRV2::GOI* constructs were transformed to *Agrobacterium tumefaciens* strain GV3101 by electroporation. Transformed agrobacteria were inoculated on LB agar selection media and cultured in the LB liquid media with antibiotics. Constructs were confirmed by PCR and used for the agro-infiltration.

### Agrobacterium infiltration and disease testing

Parental lines grown in greenhouse chambers (Unifarm, Wageningen UR, the Netherlands, under 16h light/8h dark photoperiods with 21°C/19°C day/night temperature in relative humidity of ~75%) were used for infiltration. TRV constructs were agro-infiltrated as described previously (Deng et al., 2012). Briefly, flower stem (scape) was scratched for around 1 cm length and covered with an *Agrobacterium*-soaked cotton pad. To test the Botrytis infection on TRV-treated gerbera plants, flowers were harvested at around 2 to 3 weeks post agro-infiltration. Ray florets (in the sector right above the scar due to the scratching) from each inflorescence were collected for Botrytis inoculation and for total RNA isolation.

To analyze the efficiency of CG silencing, cDNA was synthesized for RT-qPCR. Target gene and reference gene (*GAPDH*) were amplified using the cDNA of *TRV2::GOI* plants and *TRV2::GUS* plants. The change in expression of the target gene in silenced plants was normalized to the reference gene (*GAPDH*) relative to control plants (*TRV2::GUS* plant in this experiment) and represented as fold change. The method is called *2^-ΔΔCT^* (Livak and Schmittgen, 2001), where *ΔΔCT* = (C_T, Target_ - C_T, GAPDH_) *_TRV2::GOI_* - (C_T, Target_ - C_T, GAPDH_) *_TRV2::GUS_*. Three ray florets from different silenced inflorescences and three technical replications for each treatment were used.

For Botrytis resistance testing, single fresh ray florets were inoculated with 2µl of *B. cinerea* (strain B05.10) spore suspension (5×10^5^/ml). From individual inflorescences, for each treatment 5-6 ray florets were collected for the inoculation. Lesion sizes on inoculated ray florets were obtained 24 hours post infection from images using ImageJ. Data from different gene silenced treatments were analyzed by one-way ANOVA in SPSS (Version 21).

## Results

### Selection of candidate genes and homologues of gerbera

A list of 71 genes was identified from literature as potential CGs for Botrytis resistance in gerbera (**Table S2**). These CGs belong to genes affecting cell wall composition, signal transduction or secondary metabolism. Most of the CGs with confirmed involvement in Botrytis resistance were derived from Arabidopsis, grapevine and tomato being the most studied plants. Some genes in the phenylpropanoid and flavonoid biosynthetic pathway derived from the Asteraceae family, like sunflower, Artemisia and gerbera itself, were also included in the CG list as they are important for flavonoid phytoalexin accumulation during Botrytis infection.

For all potential CGs the corresponding homologous sequence of gerbera was searched for in the *Gerbera ESTs database* (Fu et al., 2016). At least one hit could be found for all CGs in the Gerbera ESTs, the contigs with the highest hit score, lowest e-value and highest identity percentage were identified as the homologous CG sequence (**Table S2**). Over half of the identified homologs (42/71) were with an e-value < 1E-180 and the identities of most of the contigs (64/71) are above 50%. Frames showing the longest ORF were used for re-BLAST analysis. Identified gerbera CG sequences compared (blastp) against the NCBI database, showed hits to annotated CG with even higher hit scores (**Table S2**). In general, almost full-length gerbera ORFs were identified based on the coding regions of other species.

### Genotyping by HRM analysis, mapping and co-localization

Primers were designed to flank a targeted SNP in the CG ORF region with expected PCR products sizes of 80-150 bp. Only primers amplifying single band amplicons were chosen for further analysis (**Table S3** and **Figure S1**). Primers designed on 29 CGs with clear grouping results in the parents test were used for genotyping the whole population and homoduplex (from homozygous SNP) and heteroduplex offspring (from heterozygous SNP) could be easily distinguished by the change in the normalized melting curve (**Figure S2**).

All 29 tested CG markers showed the expected 1:1 segregation ratio. The difference in phenotypic means in Botrytis infection disease score on whole inflorescence, bottom and ray floret test, respectively, between the two genotypic groups from these CG markers was tested by Student’s t-tests for significance. Seventeen CG markers showed a significant difference in at least one test at the level of ≤ 0.05, of which nine showed an even higher threshold value (≤ 0.01 or ≤ 0.001). Interestingly, the CG marker for *CHI* showed a highly significant association (P≤ 0.01) in both populations for whole inflorescence (**Table S4**).

**Figure 1:**
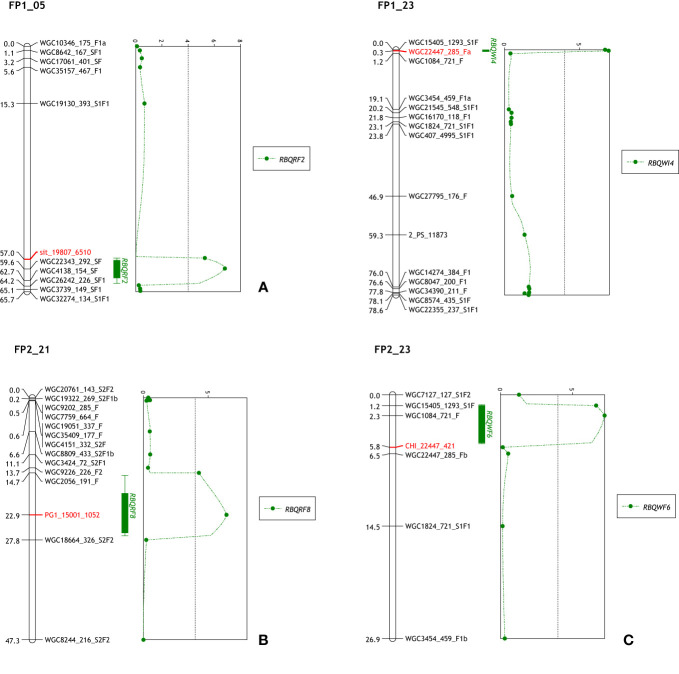
Mapped CGs and QTL positions. **(A)** *ghsitiens* mapped on FP1_05 in the region of QTL RBQRF2. **(B)** *ghPG1* mapped on FP2_21 in the region of QTL RBQRF8. **(C)** The QTLs RBQWI4 and RBQWI6 in the maternal and paternal LG23 of F population. The marker WGC22447_285_Fa is a KASP marker from contig22447 and CHI_22447_421 is a marker from the same transcriptome contig used in HRM for validation of contig.

All 29 CGs could be mapped to at least one of the linkage maps, and seven CGs (*ghCHI*, *ghDND*, *ghPER21*, *ghPG1, ghPG10, ghsit*, and *ghSS*) mapped to linkage maps in both populations (**Table S4**). CGs *ghsit*, *ghPG1* and *ghCHI* mapped in previously detected QTL regions being RBQRF2 on LG5, RBQRF8 on LG21 and RBQWI4/6 on LG23 (**Table 1** and **Figures 1A-C**), respectively. CGs *ghPG9* and *ghcutin* mapped on the paternal linkage map SP2_02 close to markers WGC23656_151_S1F1 and WGC11243_647_S2F1. On the maternal linkage map SP1 QTL RBQB1 was located between these two markers (**Figure S3**). Also CGs *ghPER62* and *ghSS* mapped in close proximity to QTLs; RBQRF7 on LG18 and RBQB2 on LG 16, respectively (results not shown).

**Table 1:** CGs co-localized with QTLs regions.

CGs	CGs marker	Mapped inside QTL region	Maps near to QTL region
*ghsit*	sit_19807_6510	RBQRF2	
*ghPG1*	PG1_15001_1052	RBQRF8	
*ghCHI*	CHI_22447_421	RBQWI4/RBQWI6	
*ghPG9*	PG9_25150_888	RBQB1/RBQB8	
*ghcutin*	cutin_4918_3081		RBQB1 (2.6 cM)
*ghPER62*	PER62_31925_540		RBQRF7 (4.9 cM)
*ghSS*	SS_5198_1668		RBQB2 (4.98 cM)

Compared with the previous QTL analysis (Fu et al., 2017), a number of small differences were detected (**Table S5**). First of all, two new QTLs were detected, RBQB7 in SP1_06 (LOD = 5.33) with CGs *ghPG7* and *ghPG10* mapped in the vicinity; and RBQB8 in SP2_02 with CG marker (PG9_25150_888) from *ghPG9*. This latter QTL was at a similar position as the previous identified QTL RBQB1 on the S maternal map (SP1_02). CG marker PG1_15001_1052 from *ghPG1* was mapped on linkage group FP2_21 in a 13 cM marker interval (Fu et al., 2017) and re-analyzes of the QTL lead to a shift of the location of the maximum LOD score to this position. Variance explained by this QTL (RBQRF8) increased from 8.0% to 10.7%. Adding CG marker PER62_31925_540 showed that the LOD score of the previous detected QTL RBQRF7 (4.09) was just a fraction below the GW threshold (4.1).

### Allelic diversity of candidate genes

Using a single bi-allelic SNP marker, CG alleles having a positive contribution on the QTL can only be identified from the parent in which the particular marker was polymorphic. To have a better understanding of the variation within genes, allelic diversity was assessed for the four parents for the CGs indicated (**Figure S4**). As a diploid heterozygous crop, at most 8 haplotypes/alleles per gene can be expected in the four parents. Allelic diversity ranged from at least 3 haplotypes (*ghSS*) to at most 7 haplotypes (*ghPG9*) in the CGs loci of the four parents. In all analyzed CGs, unique haplotype(s) which were not shared with other parents existed. In two CGs (*ghPER62*, *ghPG9*), the unique haplotype was linked to the resistance effect. The seven CGs loci from parent FP1 are all heterozygous, SP1 has six heterozygous loci (except *ghcutin*) and FP2 five heterozygous loci (except *ghsit* and *ghcutin*), while in SP2, only three loci contained two distinctive alleles in the CGs loci (ghPG9, *ghPER62* and *ghcutin*).

More than half of the SNPs were present in the third base of the codon. Overall, 57% of the SNPs were synonymous SNPs. Two alleles from *ghPER62*, *ghSS*, and three alleles from *ghcutin, ghPG9* encoded the same protein (**Figure S4**). Interestingly, a 36 bp insertion–deletion (InDel) was found in the first intron of *ghsit*, while a 1 bp InDel in the second exon region was found for an *ghCHI* allele which would lead to an early stop codon.

### Expression of candidate genes

Expression levels of *ghsit*, *ghPG1*and *ghCHI* were analyzed on ray florets. In the initial experimental setup, inoculated ray florets were sampled for expression analysis at 0 hpi, 6 hpi, 12 hpi, 24 hpi, 36 hpi, 48 hpi and 72 hpi. Spores of Botrytis already germinated on ray florets 6 hpi (**Figure S5**). The initial stages of Botrytis infection resulted in necrotic lesions which were clearly visible on ray florets and then lesions expanded quickly from the initial necrotic lesions to the whole ray floret. Expression of gerbera house-keeping gene at later stages of infection (36 h, 48 h and 72 h after inoculation) was already absent because florets had become necrotic. So final expression analyzes were performed on ray florets at 0, 6, 12 and 24 hrs after inoculation, with a control sample with mock inoculation sampled after 24 hrs (**Table S6**).

Expression of all three CGs was detected in the four parents except for FP1 for which no expression of *ghPG1* and *ghCHI* was found. The expression pattern of studied CGs showed variation at different time points but with significant up-regulation after Botrytis infection. In general, gene expression levels reached their peak at 12h or 24 h after Botrytis inoculation. There was no significant difference in gene expression between time point 0h and 24h in the mock samples.

### VIGS construction and indications for successful silencing of reporter genes

To characterize the function of CGs *ghsit* and *ghPG1*, we constructed a TRV-based VIGS system (Liu et al., 2002) which carried the target CG fragments in *pTRV2*, to suppress the level of plant endo-gene expression. Fragments of *PDS* (phytoene desaturase) and *CHS* (Chalcone synthase) that both are widely used as reporter genes to recognize the silenced phenotypes in gerbera (Deng et al., 2014; Deng et al., 2012) and other crops, and a β-glucuronidase (*GUS*) gene fragment were also cloned into the TRV2 vectors for control experiments.

Target gene fragments with lengths varying from 274 bp to 428 bp were amplified from parental gerbera cDNAs. Furthermore, a fragment with a length of 722 bp combining two gene fragments (*ghPG1* 428 bp + *ghsit* 294 bp) was developed by overlapping PCR. To confirm that fragments might not trigger any other unexpected non-target gene silencing; entire fragments of CGs and reporter genes were divided into a series of continuous 20 bp sub-sequences that were used as queries for BLASTn. In BLAST output, all 20 bp-subsequences of target genes only aligned to the original contigs indicating no other off-target hits were found that could lead to possible non-target gene silencing based on the currently available transcriptome information. Since only a few SNPs were found between the target gene fragments in the four parents, target gene fragments from SP1 were used for subsequent TRV-VIGS vector construction. All entry vectors and TRV2 constructs that were generated in the Gateway reactions have been sequenced and confirmed carrying the right target gene fragments.

Reporter genes (*ghPDS*, *ghCHS1* and *ghCHS4*) were used to identify in which sectors of the inflorescences the silencing phenotype was present. Since *ghCHS1* and *ghCHS4* silenced plants did not give visible indications of gene silencing, they were not used in further tests. TRV2 constructs with the *GUS* gene fragment were used as negative control.

In *TRV2::ghPDS* silenced plants of SP2, a visible color change emerged above the scar by scratching for agro-infiltration and along on the green trunk of the elongated scapes until the bottom of the flower head that could be easily followed (**Figures S6A-C**, as the arrows shown). Three of the six PDS silenced SP2 plants exhibited a changed pink color in ray florets of the inflorescences with varying ray floret numbers (**Figure S6D**). The ray florets from the original white inflorescences that were on sectors of the same side of the scar from the scratching and also the sectors of the opposite side of the scar turned to pink. Those pink ray florets/sectors defined in which parts of the flower head *PDS* had been silenced. The phenotype of PDS silencing in SP1 plants varied, showing a diluted orange color in parts of the inflorescences (**Figure S7A**) or few petals (**Figures S7B, C**) on the scratching side, or bleaching on ray florets (**Figures S7D-F**). Quite a few flower scapes of FP1 broken at scape scratching position as the stem extended (**Figures S8A, B**) and few even fell off, before the flower blossoming. Out of the scapes without stem breaking after flowering, no detectable phenotype was found in the reporter gene silenced inflorescences indicating that gene silencing in FP1 was likely ineffective.

### Gene expression in silenced ray florets and disease testing on candidate gene silenced ray florets

To quantify the suppression of the candidate and reporter gene expression after VIGS, we used a relative quantification method to determine the efficiency of silencing. The relative expression of target gene in negative control *TRV2::GUS* plants were set on 1. The change in expression of target genes was normalized to the reference gene (*ghGAPDH*) relative to *TRV2::GUS* silenced plant and represented as fold change.

**Figure 2:**
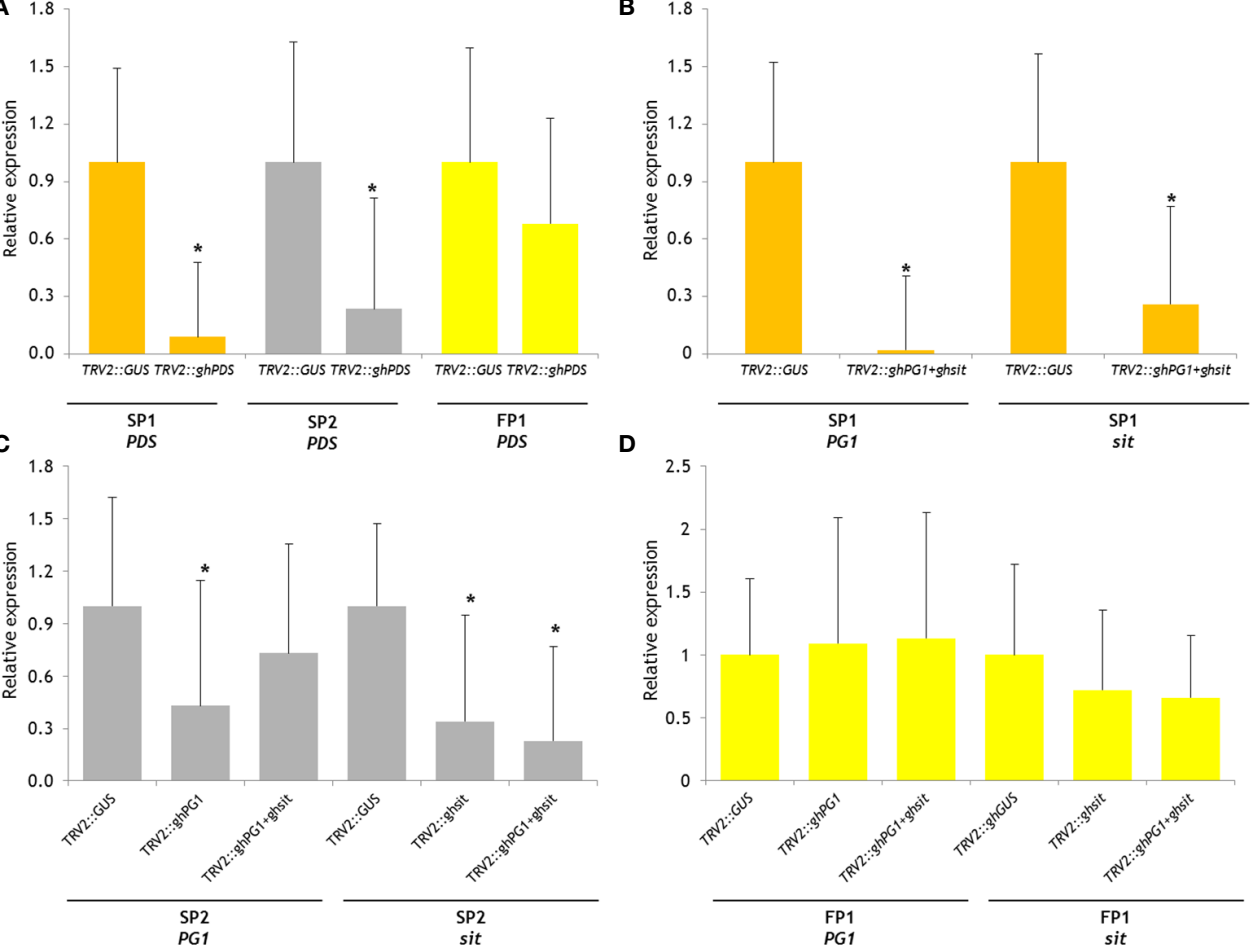
Relative gene expression level of reporter gene PDS and CGs in silenced plants using *2^-ΔΔCT^* method. The change in expression of target gene in silenced plant which was normalized to reference gene (GAPDH) relative to control plant (as 1, *TRV2::GUS* plant in this experiment) and represented as fold change. The aster indicate significant difference compared with gene expression on control TRV2::*GUS* plants (P<0.05). **(A)** Relative *PDS* expression on three parental *TRV2::ghPDS* silenced plants; **(B)** relative *PG1* and *sit* expression on SP1 *TRV2::GUS* silenced plants and *TRV2::ghPG1+ghsit* silenced plants; **(C)** relative *PG1* expression on SP2 *TRV2::GUS* silenced plants, *TRV2::ghPG1* silenced plants and *TRV2::ghPG1*+*ghsit* silenced plants; relative *sit* expression on SP2 *TRV2::GUS* silenced plants, *TRV2::ghsit* silenced plants and *TRV2::ghPG1*+*ghsit* silenced plants; **(D)** relative *PG1* expression on FP1 *TRV2::GUS* silenced plants, *TRV2::ghPG1* silenced plants and *TRV2::ghPG1*+*ghsit* silenced plants; relative *sit* expression on FP2 *TRV2::GUS* plants, *TRV2::ghsit* silenced plants and *TRV2::ghPG1*+*ghsit* silenced plants. * Significant difference compared to the control TRV2::GUS plant.

The expression levels of *PDS* in *TRV2::ghPDS* plants showed a significant decrease (P<0.05) compared to *PDS* expression in control *TRV2::GUS* plants of SP1 and SP2 (**Figure 2A**). PDS silenced FP2 showed a lower but not statistically significant difference in expression. The expression of *PG1* and *sit* were analyzed in the two-gene-silenced SP1 plant, and both genes were successfully silenced, albeit to a different extent (**Figure 2B**). Significant silencing of *PG1* expression was only in SP2 *TRV2::ghPG1* plants, but not in TRV::*ghPG1*+*ghsit* plants. While the relative *sit* expression in SP2 in both TRV2::*ghsit* and *TRV2::ghPG1*+*ghsit* plants decreased significantly (**Figure 2C**). The expression levels of neither *PG1* nor *sit* were found statistical significantly decreased on any gene silencing treatment in FP1 (**Figure 2D**).

To test Botrytis resistance on ray florets after VIGS, single ray florets were collected for Botrytis inoculation. According to the results from the indicator gene, we collected 5-6 ray florets in the sectors just right above the scratching scar around or less than 1/4 of the total ray florets. Lesion sizes 24 h post inoculation, of each ray florets from target gene silenced plant and *GUS* gene silenced plants were compared. The number of ray florets sampled from each parent and each silencing treatment for disease test is shown in **Table S7**.

**Figure 3:**
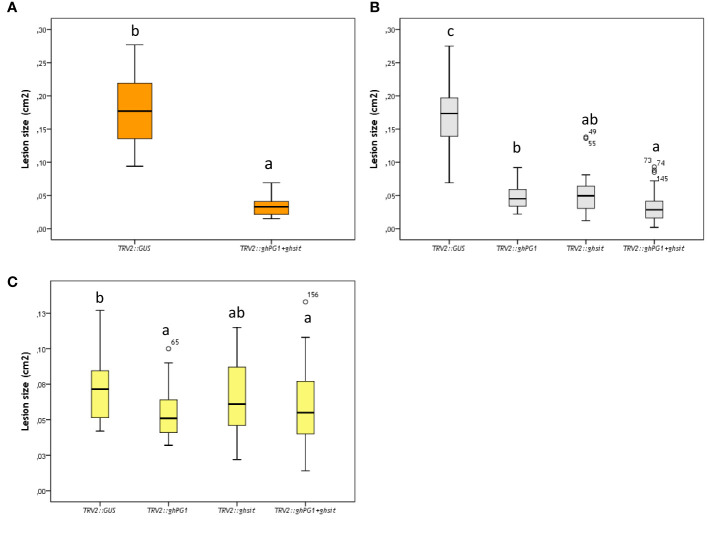
The lesion size (cm2) of Botrytis inoculation at 24hpi on different ray florets of parental silenced plants. **(A)** SP1; **(B)** SP2; **(C)** FP1. Different letters indicate significant difference (P<0.05). Every single ray floret for ImageJ measurement was coded with a number, and ray florets with number such as 49, 55, 73, 74 etc were indicated the outliers of box-plots.

Compared with the mean lesion size of control treatment (*TRV2::GUS*), the mean lesion size on ray florets from TRV2::*GOI* silenced plants showed a decrease on the three genotypes used (**Figures 3A-C**). Due to the material limitations, SP1 was only used for two genes (*TRV2::ghPG1+ghsit*) co-suppression. The lesion sizes on *TRV2::ghPG1+ghsit* co-silenced plants were reduced by 80% compared to *TRV2::GUS* silenced plants. The same amount of reduction (81%) was also observed in SP2 with *TRV2::ghPG1+ghsit* co-silencing. There were significant differences (P<0.05) between *TRV2::ghPG1+ghsit* and *TRV2::ghsit* silenced plants compared to *TRV2::GUS* silenced SP2 plants, while no significant difference between *TRV2::PG1+sit* co-silenced plants compared to *TRV2::PG1* silenced plants was found. Although the lesion size on FP1 ray florets showed less reduction and more variation than of other parents, it seems a bigger lesion reduction in FP1 came from the *TRV2::PG1* silenced plants. Significant differences could be observed in the mean of lesion size between the four treatments of FP1, yet in each treatment, large variations existed.

## Discussion

### Genotyping, mapping and the co-localization with QTLs

In this study, we selected putative CGs involved in plant resistance against Botrytis from literature and screened a gerbera EST database (Fu et al., 2016) for homologous genes. After alignment of the homologous gene sequences found among the parents and selection of suitable SNP markers, CGs were mapped on the genetic maps of two populations used for QTL mapping previously (Fu et al., 2017).

For good HRM results several criteria need to be satisfied in order to generate amplicons with just single SNPs producing easy to distinguish single-base differences on the melting curve. In such cases, all homozygotes show a single melt transition whereas heterozygotes produce a deviating melting curve arising from the integrated melting curves of two homoduplexes and two heteroduplexes (Gundry et al., 2003; Reed and Wittwer, 2004). First, PCR conditions must be optimized and primers checked to amplify a single PCR fragment, as non-specific bands can significantly reduce HRM performance (Lehmensiek et al., 2008). For 14 CG primers (12 in both populations and 2 in one population) multiple bands after amplification have been found. It might be mainly because these genes, such as *DELLA* and *CHS*, belong to gene families with homologues genes existing in the genome.

Another criterion is the amplicon size. Product size should be below 300 bp and preferably much smaller to keep a high sensitivity and specificity to detect the possible heterozygotes by HRM without error (Reed and Wittwer, 2004). When product length increases, the difference between homozygote and heterozygote curves will become smaller making SNP calling more difficult (Reed and Wittwer, 2004). Moreover, gerbera is a highly heterozygous ornamental crop and if the target sequence is larger the potential for inclusion of other unexpected SNPs is increasing. Amplicons with several SNPs always result in a complex situation with several melting curves. According to our previous study (Fu et al., 2016), there is a SNP in every 200~250 bp in each parental genotype. Thus, we produced small fragments to avoid additional SNPs.Primers were designed taking into account intron exon structure from the homologous gene coding sequence and avoiding additional SNPs as detected in the gerbera EST database.

In total, we designed 89 pairs of primers for 71 CGs, and 29 CGs with a clear 1:1 segregation in the offspring were mapped in at least one population. The percentage of genes successfully mapped was 41%, other gene primer pairs dropped out mainly because of additional SNPs and multiple bands.

Out of the 29 mapped CGs, several genes are from the same gene family. For example, several poly-galacturonase genes (*ghPG1*, *ghPG2*, *ghPG7*, *ghPG9*, *ghPG10*) could be mapped. Those genes were mapped on different linkage groups, except *PG7* and *PG10* which were mapped on the same linkage group close to each other (1 cM apart).

Using a CG approach for identifying Botrytis resistance genes in gerbera seems effective. Several mapped CG alleles showed significantly difference in resistance for the traits whole inflorescence, bottom and ray floret in the two populations, and several CGs were found co-localized with identified QTLs. Few CGs showed allelic variation that gave a high significance level (P<0.01) in the t-test, but couldn’t be detected as a QTL. This might be due to the environmental conditions which could influence the power of detecting QTLs. The detection of two new QTLs after addition of specific CGs to the map also indicated that marker density may also play a role.

### Allelic variation and expression of the candidate genes

Allelic diversity was assessed mainly for genes co-localized with detected QTLs and statistically correlated with phenotypic variation. The sequence polymorphisms of these selected CGs offer a glimpse of the heterozygosity of gerbera. Although only two species are considered to be involved in the origin of modern gerbera cultivars with a possible bottleneck at the moment of hybridization (Hansen, 1999), genetic diversity is relatively high in gerbera germplasm. The SNP density in the specific genes involved in Botrytis resistance varied from 5.7 to 27.2 SNPs/kb which is higher than the average SNP density identified within the four parental overall ESTs (from 3.7 ~ 4.8 SNPs/kb) (Fu et al., 2016). It is also higher compared to the SNP density of 10.5 SNP/kb found in the exons and introns of 7 genes in eleven safflower (*Carthamus tinctorius*) individuals (Chapman and Burke, 2007).

Multiple alleles existed at the candidate loci of the (four) parental genotypes and all genotypes are unique. Acquaah (2012) implied that for the improvement of cross-pollinated species breeding has to focus on increasing the frequency of favorable alleles. QTL analysis from bi-parental populations of gerbera in our previous study (Fu et al., 2017) only indicated the favorable segregating alleles present in our populations. Considering the heterozygous and heterogeneous situation in the four parents, it could be a practicable start to screen possible alleles focusing on these CGs in a broader gene pool and linking these to botrytis resistance.

The upregulation of expression levels of the CGs upon Botrytis infection, given the function of these genes in other species, is a clear implication that these CGs are involved in Botrytis response in gerbera. Tracking the Botrytis infection process on gerbera ray florets of parents till 72 hpi, we found that all the ray florets were infected eventually whereas the speed of disease development varied. For some CGs expression was not detected in SP2 and FP1 and that might be because of the genetic variation resulting in no expression or other genes playing a role in these two genotypes. All the studied CGs expressed in FP2 and the highest level of gene expression were at or before 12 hpi which was in line with this genotype FP2 having a relative high resistance to Botrytis in ray florets. A quick response of disease-related genes reaching the highest expression level as early as possible seems important to resist the attack of Botrytis.

The CG approach as used in this study in gerbera, for which crop no genome sequence is available, can efficiently pinpoint a number of potential causal genes. Whereas using QTL regions in outcrossing crops possess practical problems in the implementation for MAS, finding causal genes involved in a trait would be a major step and can also help in understanding the molecular interactions between Botrytis and gerbera.

### Possible mechanisms for Botrytis resistance in gerbera

Several CGs with statistical associations with the whole inflorescence, bottom and ray floret tests might be involved in Botrytis resistance under multiple mechanisms. Plant cuticle and cell wall are constituted as the first protective barriers to defense against Botrytis invasion (Curvers et al., 2010). *B. cinerea* secretes at least 6 genes polygalacturonases (PGs) to decompose plant cell walls (van Kan, 2006; Ferrari et al., 2003). However, fungal PGs can be inhibited by plant polygalacturonase-inhibiting proteins (PGIPs) whereas these proteins may not inhibit a plant’s own endo-PGs. Blanco-Ulate et al. (2014) suggested that *B. cinerea* might be able to manipulate plants to produce endo-PGs in order to degrade plant cell walls. From our study, two gerbera endo-PGs (ghPG1 and ghPG9) were found associated with Botrytis resistance on gerbera ray floret and bottom test respectively showing high statistical significance in the disease tests. The two candidate loci were detected as QTLs and explained 10.7% and 6.1% of the phenotypic variation. We assume *B. cinerea* might indeed be manipulating endo-PGs in gerbera plants to take the advantage of this in the infection process.

Plant hormones are considered to play an essential role in defense against Botrytis, especially the Ethylene (ET) and Jasmonic acid (JA) pathways. The ethylene responsive transcription factor (ERF) family encode proteins in disease resistance regulation pathways (Gutterson and Reuber, 2004) and their binding target sequence is the GCC box which is found in several promoters of pathogen related and ET- or JA-induced genes. Overexpression of ERF1 in Arabidopsis is sufficient to enhance tolerance to *B. cinerea* (Berrocal-Lobo et al., 2002). Also in our study, the ghERF is related to the phenotypic variation in the bottom test (P = 0.0075) and could be a promising candidate locus. Abscisic acid (ABA) signaling is believed to play an important role in *B. cinerea* resistance as shown in tomato mutant *sitiens* (Asselbergh et al., 2007; Curvers et al., 2010). ABA signaling regulated the cuticle and pectin composition which affect Botrytis resistance. The last step of ABA biosynthesis (ABA-aldehyde oxidation) in *sitiens* is blocked and leads to accumulation of trans-ABA instead of ABA (Rock et al., 1991). The difference of *sitiens* wild type allele (*sit+*) and mutant allele (*sit*) in tomato is in the deletion of intron 1 and division of exon 2 (Harrison et al., 2011). In our study, the two groups sorted by gerbera sit gene allelic variation showed significant difference in the ray floret test (at p<0.001 level) and the gene mapped in the QTL interval of RBQRF2. Interestingly, like the tomato sit allele, we found a 36 bp InDel in intron 1 of the four gerbera parental sit alleles (data not shown).

Phenylpropanoid compounds are natural secondary products which are derived from the general phenylpropanoid pathway and the consecutive flavonoid pathway. These derivatives, like anthocyanins, are known for the origin of flower pigmentation (Winkel-Shirley, 2001), but other derivates in the pathways like isoflavonoid phytoalexins are active in plant defence (Dixon et al., 2002). Enzymes in the phenylpropanoid/flavonoid pathway have been well studied (Elomaa et al., 1993; Helariutta et al., 1995; Ainasoja, 2008; Deng et al., 2014) in some crops and include phenylalanine ammonia-lyase (PAL), chalcone synthase (CHS), chalcone isomerase (CHI), dihydroflavonol reductase (DFR), flavanone 3-hydroxylase (F3H) on the main phenylpropanoid pathway, and 2-pyrone synthase (2-PS) and stilbene synthase (SS) on the branch for flavonoid production. Several of these metabolites were confirmed to be involved in Botrytis resistance (Dixon et al., 2002; Laquitaine et al., 2006; Koskela et al., 2011) or their expression was enhanced by Botrytis infestation (Blanco-Ulate et al., 2015). Our study confirms that for a number of genes they might play a similar role in botrytis defence in gerbera., CG marker SS_5198_1668, the homologous sequence of stilbene synthase from grapevine, is found to be co-localized with the bottom test QTL RBQB2. Alignment of the ORF region of this contig5198 with the GhCHS4 (AM906210.1), a gerbera chalcone synthase gene showed that the two sequences are identical. Deng et al. (2014) found that GhCHS4 is highly expressed in carpels. The bottom test is in accordance with “heart rot”, which is describing the disc florets infection by Botrytis. Interestingly, the disk florets color of SP1 which contributes to the QTL is black, while the other three parents have green/yellowish disk florets.

The polymorphisms of ghCHI in the two gerbera populations are associated with whole inflorescence test and the ghCHI gene might be the causal gene underlying the QTLs which were identified in the F population (RBQWI4, RBQWI6). After the Botrytis inoculation, ghCHI expression in SP1, SP2 and FP2 increased but no ghCHI expression was detected in FP1. Yellow-pigmented carnation, cyclamen and antirrhinum were all identified as due to the absence or reduction of CHI activity (Forkmann and Dangelmayr, 1980; Takamura et al., 1995; Ono et al., 2006). Considering that parent FP1 is yellow colored, it might also have lost its CHI activity. Interestingly, we found a SNP deletion in the two ghCHI alleles of FP1. The loss-of-function allele might contribute to Botrytis resistance in gerbera.

### Validation of candidate genes with VIGS

Two CGs, *ghPG1* and *ghsit*, which were mapped in QTL regions from the *ray floret* test were further characterized in this study. The two CGs are the homologs of the genes responsible for Botrytis infection in tomato. Polygalacturonases (*PGs*) are cell-wall-degrading enzymes and participate in tomato ripening and have been found to facilitate Botrytis susceptibility (Cantu et al., 2009). The ABA-deficient *sitiens* mutant in tomato is impaired in the last step of ABA biosynthesis (Taylor et al., 1988; Rock et al., 1991) and the mutant is more resistant to Botrytis than wild type tomato plants that have higher amounts of ABA (Audenaert et al., 2002). The *sit* mutant also accumulates H_2_O_2_ and changes cell walls timely and efficiently to resistance Botrytis infection (Asselbergh et al., 2007).

The normal function of these two genes, *ghPG1* and *ghsit*, might be beneficial to Botrytis infection, while when the gene expressions are suppressed, Botrytis might fail to take advantage of the genes for infection. Plant genes that facilitate pathogen infection are defined as susceptibility (S) genes and an exclusive S-gene list is given by van Schie and Takken (2014). These two genes are part of that list. Silencing S-genes can limit the ability of the pathogen for infection and whether our found CGs might function as S-genes in gerbera was tested in this study for *ghPG1* and *ghsit*. A remarkable reduction of lesion size was found in these S-gene silenced gerbera ray florets and resulted from slowing the spreading of Botrytis on ray florets for 24hpi. Denby et al. (2004) considered that the variations in lesion size on Arabidopsis ecotypes for Botrytis infection were caused by either time prior to lesion initiation or the lesion’s growth rate. Suppression of these two genes might postpone the lesion initiation and presented a delayed invasion at 24hpi on the ray florets. The two genes interacted with Botrytis in a somehow similar way which might be the reason that no further decrease of the lesion size on SP2 for the two gene silenced constructs was found compared with *TRV2::ghsit* alone.

Based on the visible indications from *TRV2::PDS* gene silenced plants of SP2, there may only be a small part, of the ray florets on the flower inflorescence be silenced. We also constructed TRV2 vectors carrying the CG *ghCHI* that mapped in a Botrytis resistance QTLs from whole flower test. However, when we sprayed the Botrytis spore on the whole flower, no visible difference was found (data not shown). The phenotypes after Botrytis infection may be difficult to detect as whole inflorescences are sprayed for the Botrytis test, but only a small part of the ray petals are silenced based on the reporter gene. To explore the function of CGs that were found with the whole inflorescence or bottom test, a stable transformation may be needed for confirming their role in Botrytis resistance.

This study confirmed that *ghsit* and *ghPG1* are involved in variation of Botrytis resistance on gerbera. After 24h of Botrytis inoculation on ray florets, a significantly delayed spread of lesions was observed on CG silenced ray florets compared to controls. For crops without genome sequence, using a CG approach could be an efficient method to pinpoint possible causal genes. VIGS here provided a rapid way to study the relationship between gene expression and susceptibility to Botrytis. Using markers developed from the causal genes themselves could make marker-assisted selection more accurate and can avoid the risk of loss of linkage due to recombination when using more distant markers. Further research will have to show the value of the combined effects of the best alleles for the two genes *ghsit* and *ghPG1* in gerbera with respect to Botrytis incidence and resistance under normal greenhouse and postharvest conditions.

## Data availability statement

The original contributions presented in the study are included in the article/**Supplementary Material**. Further inquiries can be directed to the corresponding author.

## Author contributions

YF conducted the study, and generated and analyzed data. YS was involved in the construction of the VIGS constructs. JT supervised progress and was involved in discussions. RV and PA initiated the research and did conceptualization and supervision including discussion on methodology. YF and PA wrote the original draft. YF, JT, RV, and PA reviewed and edited. All authors contributed to the article and approved the submitted version.
