# Supplementary material for: The use of a candidate gene approach to study Botrytis cinerea resistance in Gerbera hybrida
Source: Front Plant Sci. 2023 Mar 22;14:1100416. doi: 10.3389/fpls.2023.1100416 (PMC10073661; doi:10.3389/fpls.2023.1100416)
Supplement: Supplementary file 1 [file DataSheet_1.docx]

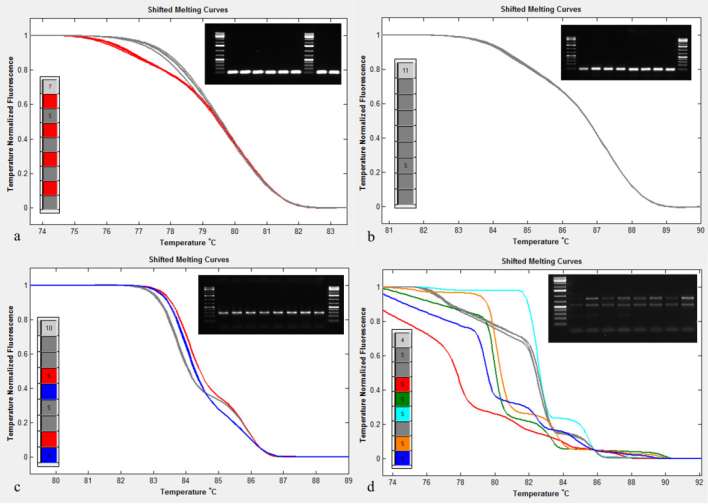


Fig. S1. Four possible cases of amplicon HRM results in parent samples. a: (contig12863/*ghPER21* gene): the PCR product size was exactly the same with what it designed and the two parents can be clearly grouped via the HRM result; b: (contig28711/*ghPgD* gene): the PCR product size as expected but the parental melting curves overlapped, cannot be used for offspring genotyping; c: (contig28693/*ghLOX* gene): Single band on the gel and the size was higher than expected, but the melting curve showed a swing line which means at least two SNPs in this region. In this case, the melting curves in progeny would be demonstrate a more complex situation (data not shown); d: (contig29198/*ghDELLA* gene): Multiple bands and also possible multiple polymorphic loci existed, not suitable for HRM analysis. The coloured eight grids on the left of each plot from the bottom to top represented the samples FP1, FP2, SP1, SP2, FP1, FP2, SP1, SP2, respectively, and the top right of the each plot is the PCR products of the parental samples on the agarose gel.


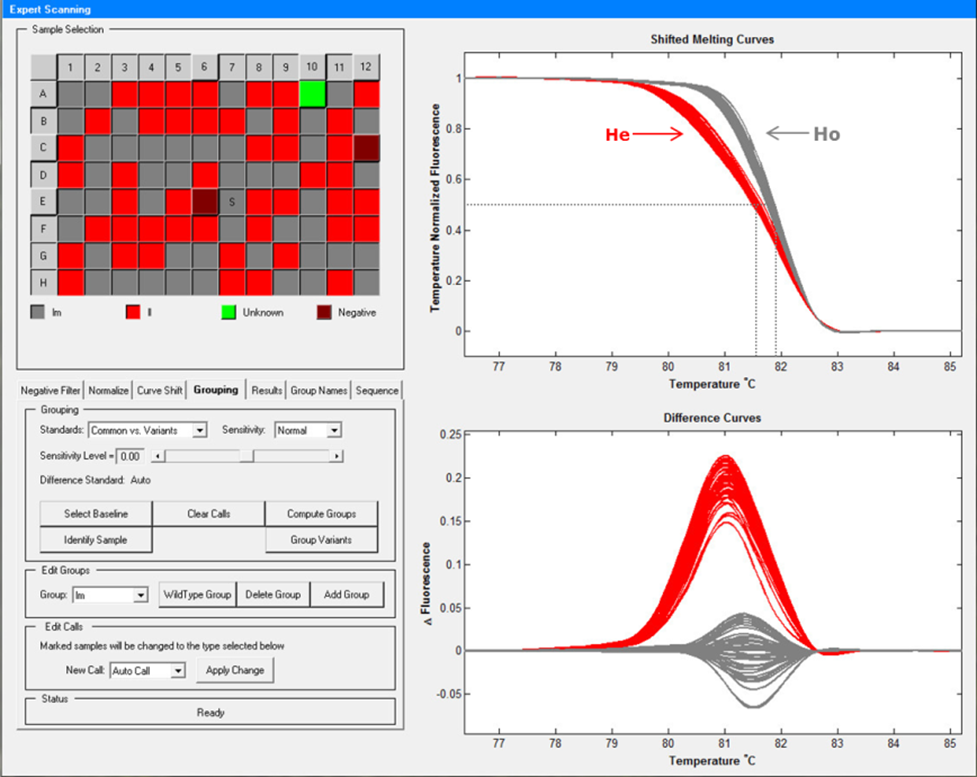


Fig. S2 Example of easily scorable HRM results: SNP results from contig1926 (the homologue hit of anthranilate synthase/AS gene in Arabidopsis) on part of the Schreurs population. Grey curve group represents homozygous alleles; red curve group represents heterozygous alleles. Note: H11 and H12 on the 96-well plate represent Parent 1 and Parent 2 (of population S), and the others represent individuals in the population. He. Heterozygote. Ho. Homozygote

Fig. S3 Mapped CGs and QTL positions. *ghPG9* and *ghcutin* mapped on the SP2_02 close to WGC23656_151_S1F1 and WGC11243_647_S2F1 which markers are withing the QTL RBQB1 region on SP1_02.


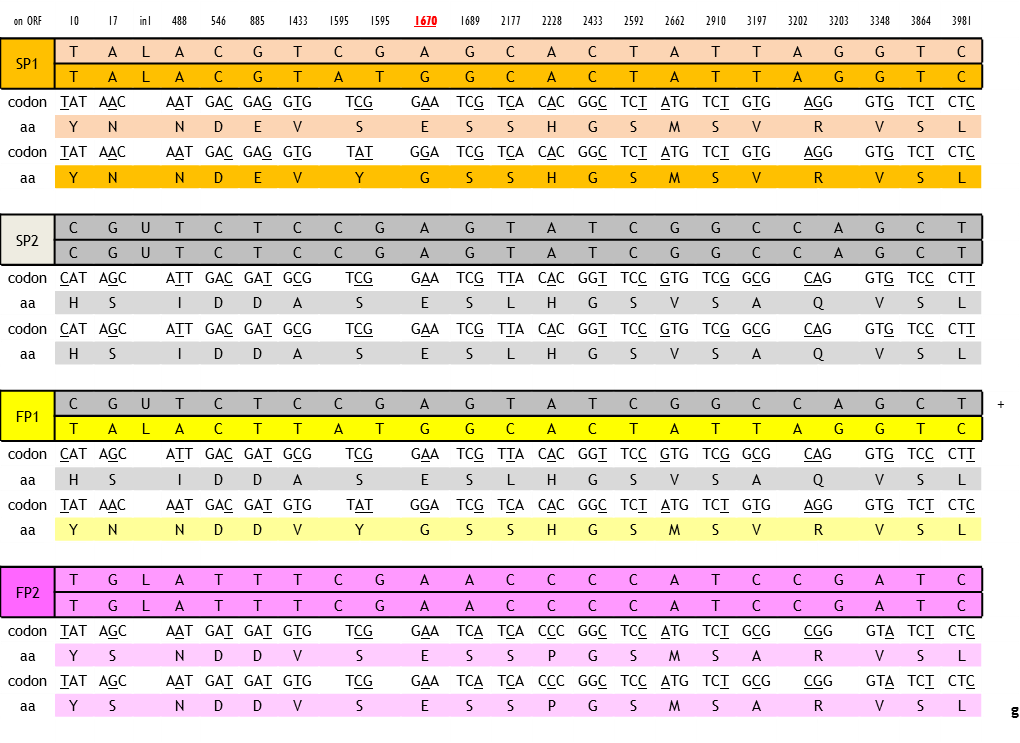
a


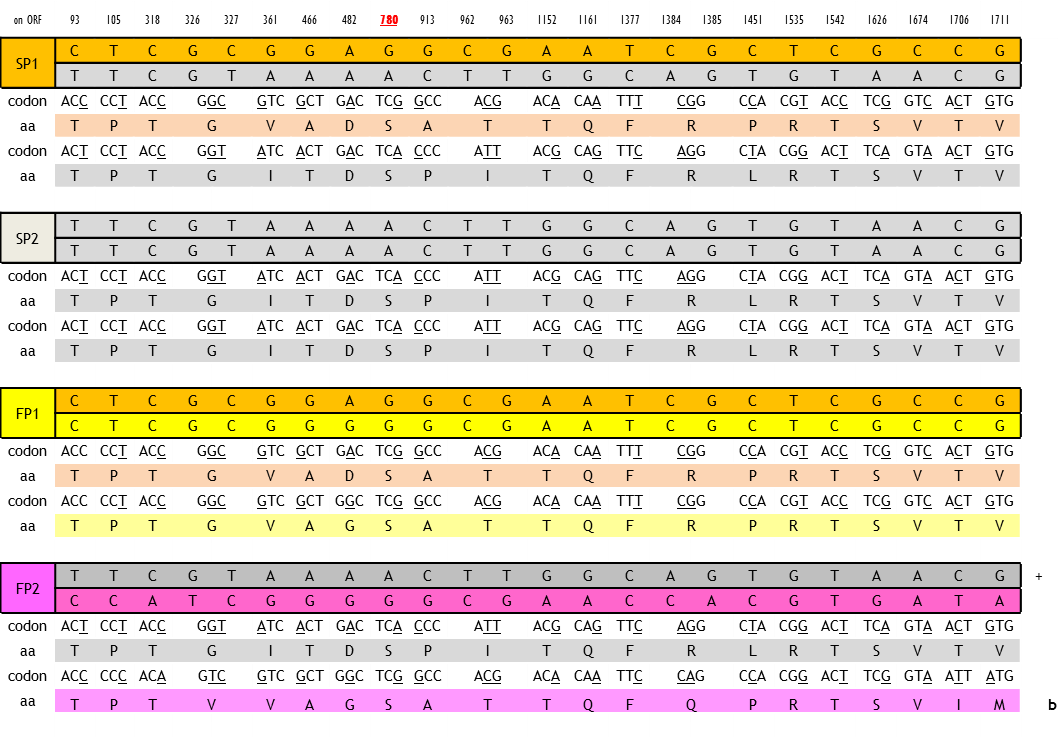
b


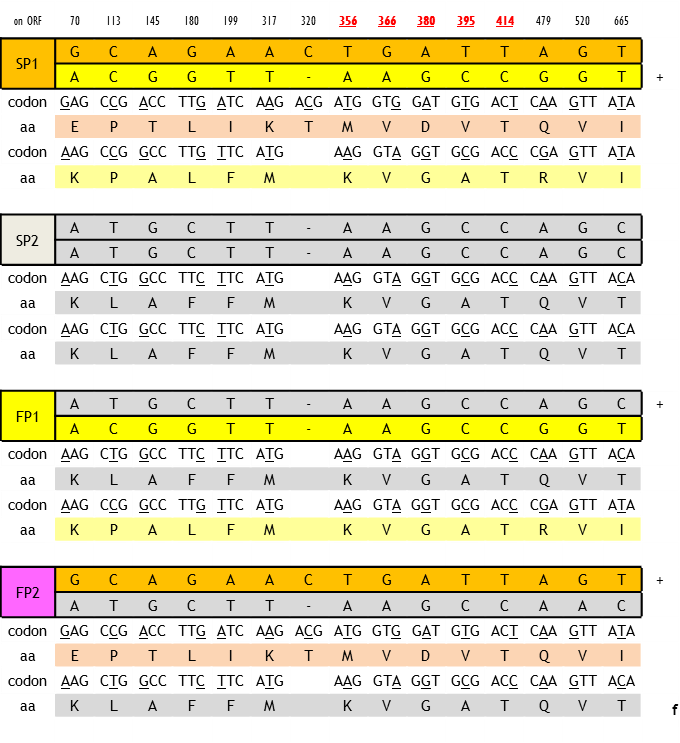
c


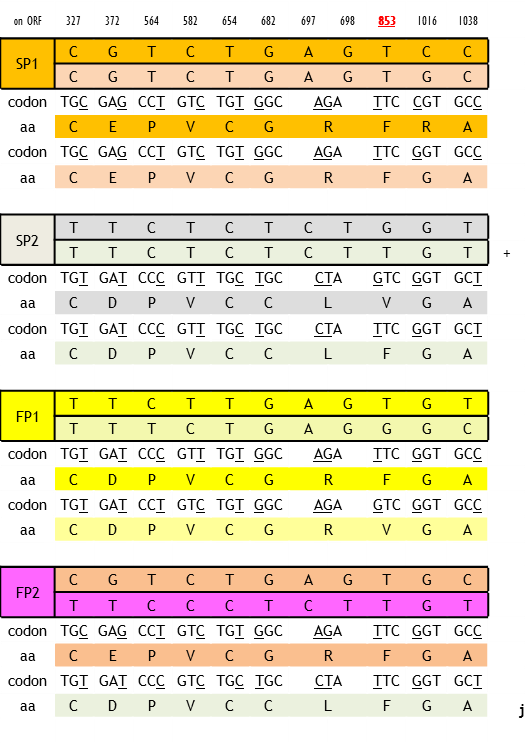
d


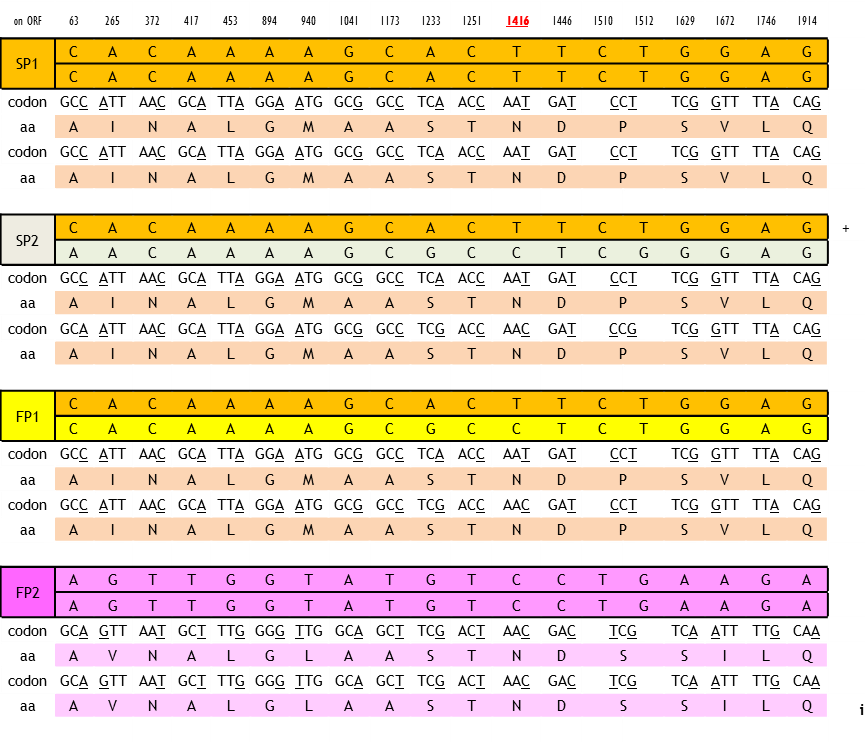
e


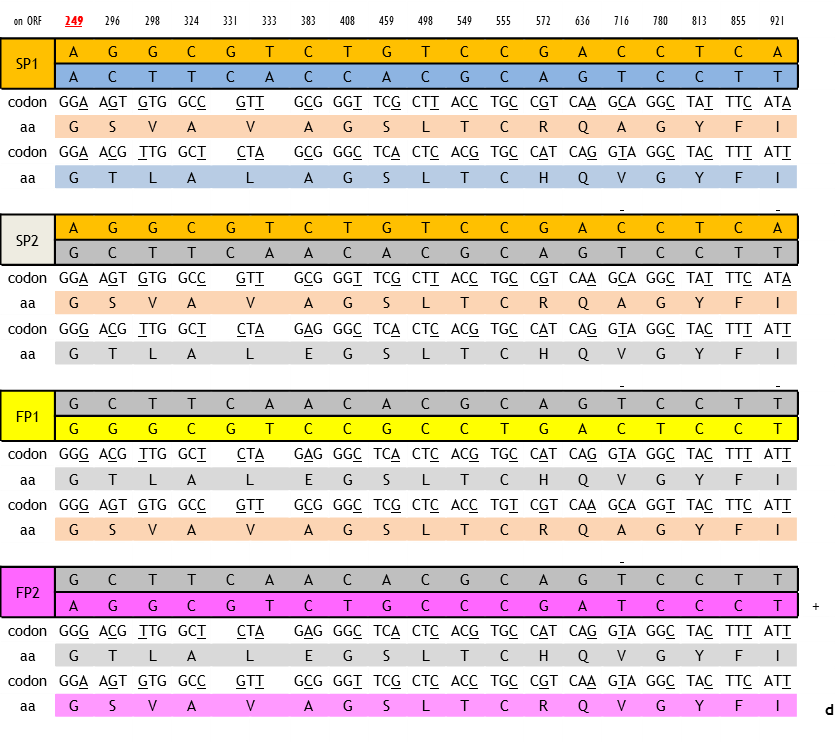
f


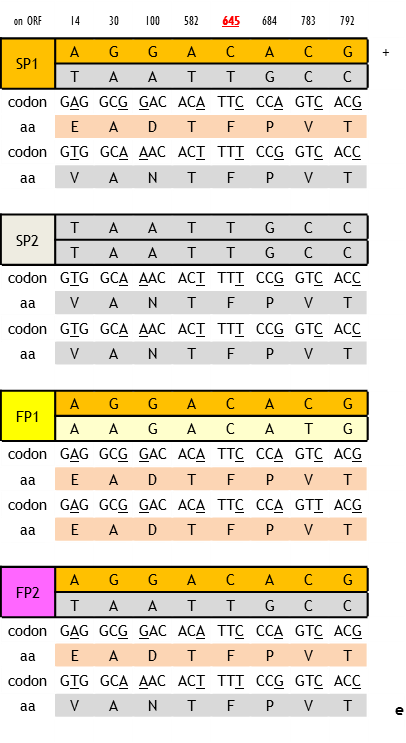
g

**Fig. S4 Alignment of CG alleles in four parents**. Allele of each gene from parents are represented only by SNP variant positions. SNP for HRM is highlighted with red and underlined. Allelic variantion of a. *ghsit*; b. *ghPG1*; c. *ghCHI*; d. *ghPG9*; e. *ghcutin*; f. *ghPER62*; g. *ghSS*;


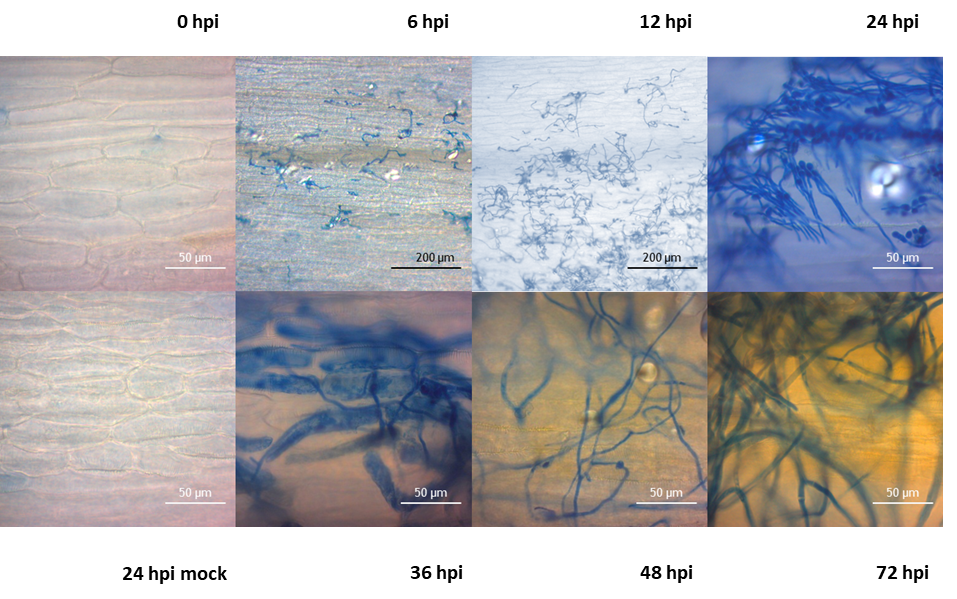

Fig. S5 Progress of disease in Botrytis-inoculated gerbera SP2 ray florets after trypan blue staining at different timepoints. At the 6hpi, the spore are germinated and hyphal growth is visible on ray florets. In 36, 48, 72 hpi, background shows that ray florets are necrotic brown.

Fig. S6 The phenotypes of SP2 plants after agro-infiltration with *TRV2::ghPDS* constructs. The crosses in (a) and (b) represent the position of scratching and the scar still remain with scape extension. The arrows on (a), (b), (c) track the potential transmission of the virus, from the scar left till the bottom of the flower head. Three gerbera flowers in (d) indicate the color changed sectors in the whole gerbera inflorescences and the number of pink ray florets is varied. SP2 basal color of the ray florets is white.


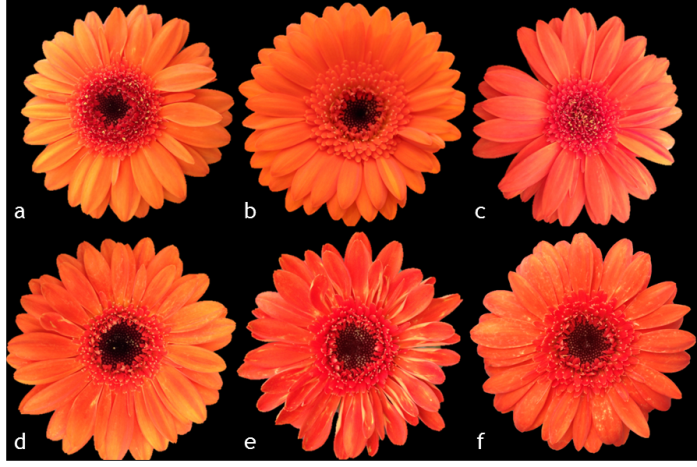


Fig. S7. The variation for *PDS* silencing in SP1 inflorescences. a, the phenotype of PDS-silencing in SP1 plants showed a diluted orange color in parts of the inflorescences; b and c, one or two ray florets with a diluted orange color; d, e and f, bleaching on ray florets.


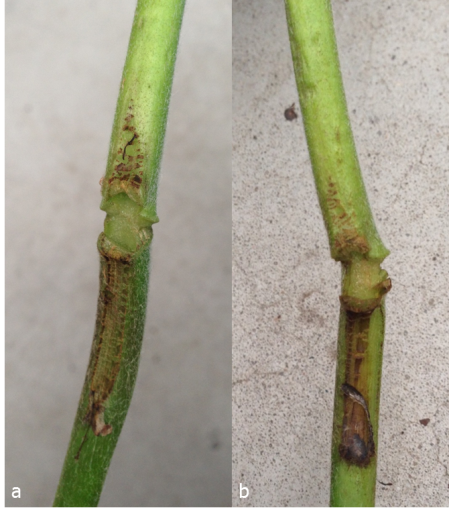


Fig. S8. Stem break on FP1. As stems extended and the flower head developed, the flower stem of FP1 broke at the site of scratching for agro-infiltration.
