# Supplementary material for: The use of a candidate gene approach to study Botrytis cinerea resistance in Gerbera hybrida
Source: Front Plant Sci. 2023 Mar 22;14:1100416. doi: 10.3389/fpls.2023.1100416 (PMC10073661; doi:10.3389/fpls.2023.1100416)
Supplement: Supplementary file 2 [file Table_1.pdf]

Supplementary Tables

Table S1 Primers used for VIGS.

Primer name	Oligonucleotide sequence (5'-3')	note
ghPDS_B1_274_F	ggggacaagtttgtacaaaaaagcaggctAGTAAAGTTTGCGATTGGGCT	*att*B1 is underlined
ghPDS_B2_274_R	ggggaccactttgtacaagaaagctgggtAAGTCTCTCAGGTGGGTTGC	*att*B2 is underlined
ghCHS1_B1_313_F	ggggacaagtttgtacaaaaaagcaggctAATTGCGTCTATCAAGCGGA	
ghCHS1_B2_313_R	ggggaccactttgtacaagaaagctgggtAGGTGGTGCAGAAGATGAGG	
ghCHS4_B1_282_F	ggggacaagtttgtacaaaaaagcaggctTGCAGTTACTTTTCGTGGGC	
ghCHS4_B2_282_R	ggggaccactttgtacaagaaagctgggtTGAAAGGCCTCTACCAAGCT	
ghPG1_B1_294_F	ggggacaagtttgtacaaaaaagcaggctAAAGCCTCCCCACTTCTACC	AF
ghPG1_B2_294_R	ggggaccactttgtacaagaaagctgggtGCCGCTGTATCTAGCAAACC	
ghsit_B1_428_F	ggggacaagtttgtacaaaaaagcaggctCTGTCGCTGCACTGGTTATC	
ghsit_B2_428_R	ggggaccactttgtacaagaaagctgggtCAGCTGCTCCAACTTCGATC	BR
ghPG1+ghsit_BF+AR	GGTAGAAGTGGGGAGGCTTTCAGCTGCTCCAACTTCGATC	BF+AR
ghPG1+ghsit_AR+BF	GATCGAAGTTGGAGCAGCTGAAAGCCTCCCCACTTCTACC	AR+BF
*att*L1	TCGCGTTAACGCTAGCATGGATCTC	sequencing
*att*L2	ACATCAGAGATTTTGAGACACGGGC	sequencing
TRV2	GTTTTTATGTTCAGGCG	sequencing
ghPDS_qPCR_F	GTGGCCAAGTCAGGCTAAAT	qRT-PCR
ghPDS_qPCR_R	ACATAAGCATCGCCTTTGAT	qRT-PCR
ghPG1_qPCR_F	GTGGGGTTTTCTGGCTACG	qRT-PCR
ghPG1_qPCR_R	AGTATCATTCGCGCCGTTAC	qRT-PCR
ghsit_qPCR_F	TTCAACCACCTGTTTTGCAG	qRT-PCR
ghsit_qPCR_R	AATTCTGGGATTTGTTCAAGATT	qRT-PCR
ghGAPDH_qPCR_F	CCAGGAACCCAGAGGAGATACC	qRT-PCR
ghGAPDH_qPCR_R	GGAGCGGATATGATGACCTTCTTG	qRT-PCR

Table S2 Candidate genes for Botrytis resistance and corresponding homologues in gerbera

Candidate gene	Function	Accession	from	amino acids	hit	Score (Bit)	E-value	Identities	ORF size in contig	BLAST back					
										Description	Score (Bit)	Query cover	E-value	Identities	Accession
2-PS	2-pyrone synthase	CAA86219.2	*Gerbera hybrida*	**402**	contig11873	817	0	99%	**402**	2-pyrone synthase, 2-PS,	817	100%	0	99%	P48391.2
ACS	ACC synthase	AEE82992.1	*Arabidopsis thaliana*	**495**	contig17688	658	0	68%	**481**	putative ACC synthase 1	855	99%	0	84%	AAP14019.1
AF	plant defensin	NP_199255.1	*Arabidopsis thaliana*	**80**	contig29000	56.6	2.00E-10	52%	**183**	DNA/RNA helicase, ATP-de	342	98%	2.00E-107	90%	KVI04017.1
AIR12	auxin-induced in root cultures	NP_566306.3	*Arabidopsis thaliana*	**273**	contig1278	169	1.00E-47	53%	**387**	PREDICTED: cytochrome	544	94%	0	68%	XP_011089631.1
AOS	allene oxide synthase	AED94842.1	*Arabidopsis thaliana*	**518**	contig28753	602	0	62%	**479**	allene oxide synthase [Ar	746	97%	0	76%	ADZ24000.1
AS	anthranilate synthase compone	AED90916.1	*Arabidopsis thaliana*	**624**	contig1926	806	0	71%	**597**	anthranilate synthase alp	1022	97%	0	85%	OIT06829.1
BIK1	Botrytis-induced kinase	NP_181496.1	*Arabidopsis thaliana*	**395**	contig14337	496	1.00E-170	66%	**402**	protein kinase apk1b, chl	660	100%	0	80%	OIT01161.1
BOS	putative transcription factor	NP_187301.1	*Arabidopsis thaliana*	**323**	contig3562	267	1.00E-85	56%	**308**	PREDICTED: transcriptio	396	97%	5.00E-136	66%	XP_011027944.1
bre1	botrytis-resistant 1	NP_182022.2	*Arabidopsis thaliana*	**878**	contig15621	821	0	52%	**878**	PREDICTED: E3 ubiquitin-	1177	99%	0	67%	XP_010661359.1
cel1	endo-1,4-beta-glucanase	NP_001234862.1	*Solanum lycopersicum*	**489**	contig13541	782	0	83%	**501**	Endoglucanase 8 [Morus r	828	93%	0	85%	XP_010101981.1
cel2	endo-1,4-beta-glucanase	NP_001234867.1	*Solanum lycopersicum*	**501**	contig5926	650	0	68%	**505**	endoglucanase [Nicotiana	700	98%	0	67%	OIT33211.1
CHI	Chalcone-flavonone isomerase	Q42663.1	*Callistephus chinensis*	**237**	contig4923	597	0	75%	**222**	Chalcone isomerase [Saus	369	99%	6.00E-128	78%	Q8LKP9.1
CHI	Chalcone isomerase	NP_001234421.1	*Solanum lycopersicum*	**262**	contig22447	270	1.00E-57	64%	**220**	chalcone isomerase [Cam	249	87%	2.00E-80	65%	AHB32112.1
CHS3	chalcone synthase	CAA86218.1	*Gerbera hybrida*	**398**	contig1332	729	0	89%	**402**	Chalcone synthase 3 [Ger	832	100%	0	99%	P48392.1
CHS1	chalcone synthase	CAA86218.1	*Gerbera hybrida*	**398**	contig29143	517	0	100%	**259**	Chalcone synthase 1 [Ger	531	100%	0	100%	P48390.1
cutin	cutin biosynthesis; long-chain	NP_175368.2	*Arabidopsis thaliana*	**665**	contig4918	936	0	68%	**660**	long chain acyl-coa synth	1110	99%	0	79%	OIT07312.1
DELLA	DELLA protein RGA	AEC05469.1	*Arabidopsis thaliana*	**587**	contig29198	687	0	64%	**562**	DELLA 1 [Lactuca sativa]	1014	99%	0	90%	BAG71200.1
DND	cyclic nucleotide-gated ion cha	NP_197045.1	*Arabidopsis thaliana*	**726**	contig29068	952	0	74%	**699**	cyclic nucleotide gated ic	1250	97%	0	90%	AIT39758.1
EDR3	dynamin-related protein 1E	NP_567094.1	*Arabidopsis thaliana*	**623**	contig24013	1013	0	80%	**644**	dynamin-related protein	1095	99%	0	83%	OIT39247.1
EIN2	ethylene signal transduction cc	NP_195948.1	*Arabidopsis thaliana*	**1294**	contig25557	932	0	45%	**1184**	ethylene signaling protei	1171	97%	0	50%	NP_001234518.1
ELP2	elongator subunit 2	NP_175377.2	*Arabidopsis thaliana*	**838**	contig2398	1092	0	65%	**835**	elongator complex protei	1266	99%	0	72%	OIS97280.1
ERF	ethylene-responsive transcript	AED95487.1	*Arabidopsis thaliana*	**243**	contig18430	176	8.00E-53	55%	**229**	AP2/ERF domain-containi	370	99%	3.00E-128	79%	KVH91971.1
ERF	ethylene-responsive transcript	AEE76738.1	*Arabidopsis thaliana*	**218**	contig11093	186	2.00E-57	57%	**196**	AP2-ERF transcription fac	241	94%	1.00E-78	70%	AFA36063.1

Table S2 (continued)

Candidate gene	Function	Accession	from	amino acids	hit	Score (Bit)	E-value	Identities	ORF size in contig	BLAST back					
										Description	Score (Bit)	Query cover	E-value	Identities	Accession
ET	ethylene-insensitive protein	AAR08678.1	*Petunia x hybrida*	**1310**	contig14926	777	0	46%	**865**	Ethylene-insensitive 2 [C	1255	98%	0	71%	KVH93917.1
EXLA2	expansin-like A2	NP_195553.1	*Arabidopsis thaliana*	**265**	contig14980	313	2.00E-105	67%	**260**	PREDICTED: expansin-like	404	95%	6.00E-141	74%	XP_002534094.1
EXP1	expansin 1 protein	AAQ12264.1	*Solanum lycopersicum*	**261**	contig12313	423	2.00E-146	80%	**284**	PREDICTED: expansin-A4	456	91%	5.00E-161	84%	XP_008384848.1
FDH	fiddlehead protein; 3-ketoacyl-	AAF73973.1	*Arabidopsis thaliana*	**550**	contig12215	878	0	76%	**554**	hypothetical protein Ccr	1059	100%	0	92%	KVH88528.1
GDSL	GDSL-like Lipase/Acylhydrolase	NP_194743.1	*Arabidopsis thaliana*	**348**	contig11795	265	3.00E-83	45%	**360**	Lipase, GDSL [Cynara car	479	98%	6.00E-167	65%	KVI00927.1
GLK1	transcription activator Golden	NP_565476.1	*Arabidopsis thaliana*	**420**	contig15145	237	3.00E-71	46%	**411**	transcription activator g	429	100%	5.00E-145	58%	OIT00132.1
GSL5	callose synthase 12	NP_192264.1	*Arabidopsis thaliana*	**1780**	contig30332	2565	0	70%	**1756**	callose synthase 12 [Nico	2806	99%	0	75%	OIT39115.1
GST1	glutathione S-transferase	AEE27498.1	*Arabidopsis thaliana*	**208**	contig17531	149	9.00E-44	53%	**160**	Glutathione S-transferase	535	100%	2.00E-69	68%	KVI12466.1
LOX	lipoxygenase 2	AGO03786.1	*Tanacetum cinerariifoli*	**899**	contig28693	1318	0	72%	**901**	Lipase/lipooxygenase, PL	2575	100%	0	78%	KVH90900.1
LOX	lipoxygenase	AGO03785.1	*Tanacetum cinerariifoli*	**907**	contig13375	1484	0	82%	**920**	lipoxygenase 1 [Tanacetu	1550	100%	0	82%	AGO03785.1
LOX	lipoxygenase 2	AAA32749.1	*Arabidopsis thaliana*	**896**	contig4808	1011	0	59%	**829**	Lipase/lipooxygenase, PL	1369	100%	0	78%	KVI00262.1
MKP2	MAP kinase phosphate 2	NP_850522.1	*Arabidopsis thaliana*	**167**	contig427	181	4.00E-55	59%	**179**	Dual specificity phosphat	306	92%	1.00E-104	88%	KVH88866.1
MYB46	transcription factor MYB46	AED91824.1	*Arabidopsis thaliana*	**280**	contig11297	211	2.00E-63	90%	**330**	MYB transcriptional fact	332	100%	5.00E-110	54%	BAR45570.1
OPR	12-oxophytodienoate reductas	AEE35875.1	*Arabidopsis thaliana*	**372**	contig196	608	0	79%	**378**	PREDICTED: putative 12-	651	95%	0	84%	XP_017237794.1
OPR3	12-Oxophytodienoate reducta	NP_001233873.1	*Solanum lycopersicum*	**396**	contig12803	639	0	76%	**394**	artemisinic aldehyde red	720	99%	0	87%	BAU61367.1
P450	cytochrome P450 reductase	AFO64618.1	*Artemisia annua*	**704**	contig15117	1117	0	85%	**709**	cytochrome P450 reduct	1243	100%	0	86%	AFO64618.1
P450	cytochrome P450 mono-oxyger	ADO16182.1	*Artemisia annua*	**515**	contig8091	674	0	67%	**378**	cytochrome P450 mono-o	512	98%	1.00E-177	67%	ADO16182.1
PAD	auxin signaling F-box 2	NP_566800.1	*Arabidopsis thaliana*	**575**	contig12282	685	0	59%	**574**	PREDICTED: protein TRAN	981	99%	0	81%	XP_011082496.1
PAL	cytochrome P450 reductase	AFO64618.1	*Artemisia annua*	**704**	contig25584	1122	0	83%	**711**	cytochrome P450 reduct	1225	100%	0	83%	AFO64618.1
PAL	phenylalanine ammonia-lyase 1	NP_181241.1	*Arabidopsis thaliana*	**725**	contig10623	1135	0	85%	**619**	phenylalanine ammonia-ly	1205	99%	0	93%	AAL55242.1
PDF1.2	ethylene- and jasmonate-respo	NP_199255	*Arabidopsis thaliana*	**80**	contig13068	56.2	8.00E-11	52%	**123**	defensin [Helianthus ann	143	90%	4.00E-42	74%	AAM27914.1
PER21	Peroxidase 21	Q42580	*Arabidopsis thaliana*	**327**	contig12863	449	1.00E-153	66%	**324**	PREDICTED: peroxidase 2	503	99%	1.00E-177	72%	XP_007027286.1
PER62	Peroxidase 62	Q9FKA4	*Arabidopsis thaliana*	**319**	contig31925	321	1.00E-106	51%	**315**	PREDICTED: peroxidase 2	472	95%	9.00E-166	74%	XP_012078787.1

Table S2 (continued)

Candidate gene	Function	Accession	from	amino acids	hit	Score (Bit)	E-value	Identities	ORF size in contig	BLAST back					
										Description	Score (Bit)	Query cover	E-value	Identities	Accession
PFT1	polymerase II transcription sul	NP_173925.3	*Arabidopsis thaliana*	**836**	contig12433	714	0	59%	**768**	Phytochrome and floweri	948	89%	0	71%	EOY02123.1
PG1	polygalacturonase 2	NP_001185361.1	*Arabidopsis thaliana*	**626**	contig15001	642	0	58%	**622**	polygalacturonase 1 beta	812	99%	0	65%	OIT26928.1
PG10	polygalacturonase 1	AEE33681.1	*Arabidopsis thaliana*	**624**	contig11147	704	0	63%	**631**	polygalacturonase-1 non-	872	96%	0	68%	OIS96021.1
PG2	polygalacturonase	BAA89476.1	*Salix gilgiana*	**393**	contig1158	380	4.00E-126	49%	**410**	Polygalacturonase [Theol	429	96%	5.00E-146	53%	EOX93416.1
PG7	polygalacturonase	NP_001234256.1	*Solanum lycopersicum*	**452**	contig35500	375	5.00E-124	50%	**450**	PREDICTED: probable poly	678	100%	0	69%	XP_012085412.1
PG9	polygalacturonase 3	XP_002884668.1	*Arabidopsis lyrata* subs	**391**	contig25150	323	2.00E-106	50%	**347**	PREDICTED: exopolygalac	372	93%	1.00E-124	58%	XP_011003515.1
PgD	defensin	AAR84643.1	*Picea glauca*	**83**	contig28711	56.2	5.00E-11	53%	**63**	Defensin-like protein [Gly	103	100%	9.00E-28	76%	KHN06998.1
PGIP1	polygalacturonase-inhibiting pr	AIF27225.0	*Helianthus annuus*	**325**	contig17627	500	9.00E-176	80%	**329**	polygalacturonase-inhibit	536	96%	0	80%	AIF27224.1
PGIP2	polygalacturonase-inhibiting pr	AIF27225.1	*Helianthus annuus*	**330**	contig20957	440	2.00E-146	70%	**329**	polygalacturonase-inhibit	521	99%	0	78%	AIF27227.1
PLDB1	phospholipase D beta 1	NP_565963.2	*Arabidopsis thaliana*	**1083**	contig14327	1344	0	79%	**1091**	phospholipase d beta 1 [N	1408	99%	0	65%	OIT39456.1
PLP2	phospholipase A 2A	NP_180224.1	*Arabidopsis thaliana*	**407**	contig6394	575	0	69%	**444**	Phospholipase A 2A, IIA,Pl	617	90%	0	74%	EOY14528.1
PME3	pectin methylesterase 3	NP_188048.1	*Arabidopsis thaliana*	**592**	contig28400	772	0	72%	**585**	pectinesterasepectineste	941	100%	0	75%	OIT26967.1
RAP	ethylene-responsive transcript	NP_850582.1	*Arabidopsis thaliana*	**375**	contig25598	214	7.00E-64	46%	**332**	AP2/ERF domain-containi	519	100%	0	75%	KVI05829.1
RD21	cysteine proteinase	NP_564497.1	*Arabidopsis thaliana*	**462**	contig20206	607	0	69%	**455**	cysteine protease-1 [Heli	757	99%	0	79%	BAC75923.1
rwa2	Reduced wall acetylation2	NP_001078116.1	*Arabidopsis thaliana*	**568**	contig29675	843	0	74%	**545**	PREDICTED: protein REDL	929	100%	0	80%	XP_004229023.1
sitiens / sit	ABA-deficient mutant/ABA aldel	ADR31354.1	*Solanum lycopersicum*	**1361**	contig19807	1727	0	67%	**1341**	Lactuca sativa aldehyde	2033	99%	0	75%	BAE72098.1
SR1	calmodulin-binding transcriptio	NP_850023.1	*Arabidopsis thaliana*	**1032**	contig1903	962	0	51%	**1080**	PREDICTED: calmodulin-bi	1427	99%	0	66%	XP_010645223.1
SS	stilbene synthase 1	NP_001267939.1	*Vitis vinifera*	**392**	contig5198	597	0	75%	**389**	chalcone synthase [Gerbe	801	100%	0	99%	CAP20328.1
SSI2	acyl-[acyl-carrier-protein] des	NP_181899.1	*Arabidopsis thaliana*	**401**	contig11878	661	0	87%	**426**	stearoyl-acyl-carrier pro	790	92%	0	94%	ABF66638.1
STP13	sugar transport proteins 13	NP_198006.1	*Arabidopsis thaliana*	**526**	contig6824	813	0	85%	**519**	PREDICTED: sugar transp	914	98%	0	86%	XP_011090738.1
TAGL1	TAGL1 transcription factor	AAM33101.2	*Solanum lycopersicum*	**269**	contig30005	281	4.00E-92	66%	**247**	MADS-box protein, GAGA1	505	100%	0	97%	CAA08800.1
WRKY33	WRKY transcription factor 33	AAM34736.1	*Arabidopsis thaliana*	**512**	contig16847	347	2.00E-111	56%	**498**	WRKY33 [Chrysanthemun	751	100%	0	76%	AJF11718.1
ZF	zinc finger CCCH domain-conta	AEC09788.1	*Arabidopsis thaliana*	**597**	contig30219	529	4.00E-180	52%	**610**	Zinc finger family protein	671	99%	0	53%	EOY17761.1
ZF	zinc ion binding protein	NP_565200	*Arabidopsis thaliana*	**358**	contig1325	172	5.00E-49	44%	**331**	Zinc finger, RING-type [C	478	100%	8.00E-168	79%	KVI10234.1
ZF	zinc finger protein	AEE30870.1	*Arabidopsis thaliana*	**277**	contig30683	172	2.00E-51	50%	**238**	Cys2/His2-type zinc fing	315	100%	5.00E-106	66%	AFF57513.1

Table S3 Primers list for candidate genes used in HRM and qRT-PCR analysis

CGs markers	Primer	HRM results in parents		note
		S	F	
2-PS_11873_814_F<br>2-PS_11873_814_R	GGCCTGAACCCACAATGAGT<br>GGACCCAATGAGAACCACCTT	b	a	*
ACS_17688_842_F<br>ACS_17688_842_R	ACCCTTTGGGCACTTTTCTT<br>CGCAAATGAGGTGGATGTTT	a	a	
AF_29000_1070_F<br>AF_29000_1070_R	CCGAGAAGTTGGCTCAAGAC<br>TTTCTCGGCTTCGACTTTGT	d	d	
AIR12_1278_1722_F<br>AIR12_1278_1722_R	ATCTAACCTCCCTTTCGCCC<br>GGAATTCCGGCGAAACATGA	d	d	
AOS_28753_1931_F<br>AOS_28753_1931_R	CTGATCTCCGACGCCAAT<br>TCCCCAATACAATCAAATGGA	c	c	
AOS_28753_2837_F<br>AOS_28753_2837_R	GTCGCTTGGAACTGTGGAAT<br>CCTCCTCTTCTTCATGCTCAA	b	d	
AS_1926_1135_F<br>AS_1926_1135_R	TCTGCACATTGTTTTTCATCA<br>CAGGAGAGGGAAGACACCAA	a	a	
AS_1926_I2_F<br>AS_1926_I2_R	CCAGGTTTAAAGGCTTCCAGT<br>AGCTCCAATAACACTGTACCG	a	b	
BOS_3562_1071_F<br>BOS_3562_1071_R	TGCATACATTATTGGATTAGGAAGA<br>TCACAAATTAGAACATGTATCTCACAA	b	b	
bre1_15621_850_F<br>bre1_15621_850_R	GCTTCTGAACTTGATCCGAACA<br>AGAGGAAGATTCAACAGGGCA	c	c	
cel2_5926_809_F<br>cel2_5926_809_R	ATTCTCCGGGAACTGAGGTG<br>AAGCTTTCTCGAGTACTTTGAATCC	c	c	
CHI_22447_421_F<br>CHI_22447_421_R	GACGGTGAAAATGGTGATTGT<br>TTGTGAGGTGGTCGTGTTTT	a	a	
CHI_22447_175_F<br>CHI_22447_175_R	GAAACGATAACCGGAAATGC<br>CCGTCGCTTAGCTTAACCAG			**
CHI_4923_1704_F<br>CHI_4923_1704_R	CCATAACTGCCGGAGTGAAC<br>GAGGTATCTAACTTTTCCTGTTCTCC	b	b	
CHS_1332_1045_F<br>CHS_1332_1045_R	ACCGGTGGTGGTCTTTCC<br>TGGTAATATGTCCAGCGCCT	d	d	
CHS_29143_1582_F<br>CHS_29143_1582_R	GGAATCAAGGTGGGTGTCAT<br>CTCGCGGAGAACAATAAAGG	a	b	**
cutin_11795_2336_F<br>cutin_11795_2336_R	GGACTTGAGGAGCACCATCC<br>TGGGTTTCGATATTAAGGTTTTG	a	a	
cutin_11795_2266_F<br>cutin_11795_2266_R	CCCATCGGGAAAGTCAATC<br>TTACTGAGGCAAAAGCCAAT	c	c	
cutin_11795_2066_F<br>cutin_11795_2066_R	AACAAGGGACTTGAGGAGCA<br>GGTTTCGATATTAAGGTTTTGATTT	d	b	
cutin_4918_2779_F<br>cutin_4918_2779_R	GTGACATGTTGCACCGTTCT<br>TGAAACATCCACCACAACTTTC	d	d	
cutin_4918_3081_F<br>cutin_4918_3081_R	GTGGCGAAATTTGCCTGAG<br>TCACGCTTGTGATACCCAGA	a	a	
cutin_6590_1039_F<br>cutin_6590_1039_R	GCTGTTCAAGCTGTTGCAGT<br>TACTCTGAGCCCAGCAATCA	b	a	
DELLA_29198_2133_F<br>DELLA_29198_2133_R	TTATTTGCAGGAAGTGGGTTG<br>TGCAACAAAACCCCTGTATTC	d	d	
DELLA_29198_2293_F<br>DELLA_29198_2293_R	ACAGGGGTTTTGTTGCAGAG<br>TGCAACAACTTCACCTTCTCTT	d	d	
DND_29068_911_F<br>DND_29068_911_R	GGTCGGTTCCACACATGTAA<br>TCGAGCCAGGAGGGTATTTA	a	a	
ELP2_2398_1249_F<br>ELP2_2398_1249_R	ATTCAGTCGAGTGGCAACCA<br>GCTTTCGGGTTGATAACACGGG	a	a	
ERF_11093_229_F<br>ERF_11093_229_R	CAAATAGTCACTACCCAAATCCTC<br>GAGAAGGAGGCTAAGTTGGAAA	a	a	
ERF_18430_480_F<br>ERF_18430_480_R	GCCGGACACCTCTGTAATGT<br>TGAGATTTCAAGCGGAATCG	b	c	
ET_14926_1910_F<br>ET_14926_1910_R	CCGATATCTCCGGGCTTT<br>CTGGACTGGGAAGACTGACC	c	a	

Table S3 (continued)

CGs markers	Primer	HRM results in parents		note
		S	F	
EXLA2_14980_495_F<br>EXLA2_14980_495_R	GGCCGGAACCCTACTTGT<br>AAGCCCTAATTATCTTGCCATCA	d	d	
FDH_12215_1158_F<br>FDH_12215_1158_R	GCCGGTTCCATCACTTTCTC<br>AACACCAAAACCAGCACCG	a	a	
GSL5_30332_2672_F<br>GSL5_30332_2672_R	CGAGTGTCACGAAGCTCATT<br>AACCGACGATCTTAGGAGTTC	c	a	
GST1_17531_312_F<br>GST1_17531_312_R	TTATCAGATCCGTCCCCTTG<br>ATTCGAATCAAGGGCGATTA	c	b	
LOX_13375_1313_F<br>LOX_13375_1313_R	GATGGCTTCCCAGATCAAGA<br>TCCTGACCCGAGTCAAAGAC	a	a	**
LOX_13375_3067_F<br>LOX_13375_3067_R	TTGCAAGGGAAATGAACAGG<br>GGAGCAATCACCGTCACTAA	a	c	
LOX_28693_4515_F<br>LOX_28693_4515_R	TGGTCTCCGTCAATGGATCT<br>GGCGATGAAAATCCTTACCC	c	c	
MMP3_15525_1055_F<br>MMP3_15525_1055_R	ACCGTACAACACCTGAATGC<br>GGAGTACGGAAAGTCGAGCT	a	a	
MYB_11297_481_F<br>MYB_11297_481_R	GGCAACAGGTGGTCTCAAAT<br>CCAGAAGTTCTTGATTTCGTTATCT	b	a	
OPR_196_1138_F<br>OPR_196_1138_R	AGGCTTTGGGTCTATACAT<br>TTTTCATCCTCGGTTC	d	d	
OPR_196_514_F<br>OPR_196_514_R	CCAGGTATATGGACAAAAGAACAA<br>CGTGAACCGCATCTACAATG	c	a	
OPR3_12803_809_F<br>OPR3_12803_809_R	CGGTGGCTTTCTCATCACTG<br>TCCAAGCCTCGACTTGTTCT	c	c	
P450_15117_2118_F<br>P450_15117_2118_R	TTCCATGCTGCGAAATCAT<br>AAAACGCCTTGTTCCTGTTG	a	a	
P450_8091_1686_F<br>P450_8091_1686_R	TGGGCCGTAGGATTTAGAGA<br>TTGCACCAGATCAACAAATCA	b	c	
PAD_12282_675_F<br>PAD_12282_675_R	ATGAAAGGCGACGAAGCTCT<br>GAACCACAAGCCCCAGACTA	a	a	
PAD_12282_1133_F<br>PAD_12282_1133_R	TGAAGTGAGCCTAGAACAAATGG<br>CCTATCAGGGTTCTGGGATG	a	b	
PAL_10623_2338_F<br>PAL_10623_2338_R	GATCGAATCACCTCAATTTGC<br>CCAAAACAAGACCGTTACGC	a	b	
PAL_25584_2562_F<br>PAL_25584_2562_R	GTCTGCTTTGATTGCCTTGG<br>AGCAGGAGATGCGAGGAAT	a	a	
PAL_28938_2050_F<br>PAL_28938_2050_R	CCTGAAAATTCCCACCGTGA<br>GGCTAGGTCCGCTTATCGAGG	a	a	
PER21_12863_774_F<br>PER21_12863_774_R	GCGAGATACAACTGAATTACTACTCA<br>AACGATTTCTTCGGCATTTG	a	a	**
PER62_31925_540_F<br>PER62_31925_540_R	ACGGACCAAATGCTGAGAAA<br>CTTCGCTTTTGCATTCTCTACT	c	a	**
PFT1_12433_1433_F<br>PFT1_12433_1433_R	TGCCGTCAAATCAGAGTCCT<br>TTTACAGTAGCGGGAGGAATG	c	c	
PG1_15001_1052_F<br>PG1_15001_1052_R	GTGGGGTTTTCTGGCTACG<br>AGTATCATTCGCGCCGTTAC	a	a	**
PG10_11147_5246_F<br>PG10_11147_5246_R	GTCGGATCGGTTGAAGACAT<br>CGTTTGTGTTGTCGGTTGTC	a	b	
PG10_11147_2266_F<br>PG10_11147_2266_R	GCCGGAAACTCAGAAGACC<br>ACGAGGGTTTCGGTAGATCA	a	b	
PG10_11147_3429_F<br>PG10_11147_3429_R	GCAGGGACCAGACGTTCAC<br>GTTTCCCTTTTTGCCGTAGC	b	a	
PG2_1158_857_F<br>PG2_1158_857_R	TTGACCAGAAGGGCAATATTCT<br>AATGCATTTCGAAGACATCGT	b	a	
PG3_10267_1039_F<br>PG3_10267_1039_R	CAATAGGGTTCCTTGAATTTCG<br>ACTACACCGGCCAAGCTTCT	b	c	
PG3_10267_143_F<br>PG3_10267_143_R	AATTTTTGCCCGTGAATGTT<br>TTAGTGGCCAATTTGTGCAT	b	b	

Table S3 (continued)

CGs markers	Primer	HRM results in parents		note
		S	F	
PG4_21627_1257_F<br>PG4_21627_1257_R	TCAAACAAGGCATGTTCAAA<br>TGAAGTTCCCTTAATCTTCACGA	d	d	
PG5_8410_454_F<br>PG5_8410_454_R	TGGTTTGCCGTTGTTTCTTA<br>CAAAAACTCAAACCGCAATG	c	c	
PG6_17218_1141_F<br>PG6_17218_1141_R	ATGGTGTCCGCATCAAAACT<br>GGTTCGTCACATTCTGCATC	b	c	
PG6_17218_605_F<br>PG6_17218_605_R	GACCGTGTTGACAACCTCCT<br>GCAGGAATTTTTCCACCAAA	b	c	
PG7_35500_1285_F<br>PG7_35500_1285_R	ACAAAGGCTGGACGGTAAAA<br>TCTGCAGACGGTTGAACTAA	a	c	
PG8_10107_508_F<br>PG8_10107_508_R	TGAGGCCGAAGTACCTTTGA<br>GCCCTTTCCAGAAATGTGAC	c	b	
PG9_25150_888_F<br>PG9_25150_888_R	CAGAGGAACTTCAGGGACGA<br>TCAAGTCCACTTCAGAAATCTCC	a	c	
PgD_28711_481_F<br>PgD_28711_481_R	TTTGGTGCAGAAACAACGAC<br>AAGACTGAAGGCTTTCAAGGTG	c	c	
PGIP1_17627_1064_F<br>PGIP1_17627_1064_R	GACGACAAACCGTATCACTGAC<br>CAAATCACCAATGGCATCAG	a	c	
PGIP1_17627_575_F<br>PGIP1_17627_575_R	TGTCACCAGTGTGTTTTCCA<br>CGATGTTATTTGGTGCATCG	d	d	
PGIP1_17627_928_F<br>PGIP1_17627_928_R	TAGACTCAAATACGTCACGTT<br>GCCCGATAGGTTGTTGAATGA	a	c	
PGIP2_20957_2153_F<br>PGIP2_20957_2153_R	TTGTACCATTGGCAGCAATC<br>CTCTCTCCCAAACCTGCAAC	d	d	
PGIP2_20957_910_F<br>PGIP2_20957_910_R	GGATCAAACAAGACGGTTCAA<br>GACTCCCGGAAATCGTGTTA	b	a	
PLDβ1_14327_2622_F<br>PLDβ1_14327_2622_R	TGATGCTGGATATGGTAAAAGAAA<br>ACCGACAAGATTACCCGTGA	c	c	
RAP_25598_2336_F<br>RAP_25598_2336_R	TTGAAGGAAACTGGGATGCT<br>GAAGGTCATCGAAGGTCCAA	c	c	
RAP_25598_2431_F<br>RAP_25598_2431_R	TCTTTGGACCTTCGATGACC<br>CCTTATTTACAAACATGAGCGAAA	a	c	
RAP_25598_705_F<br>RAP_25598_705_R	CAGCCTCCAAAGACTCTGCT<br>TTCTCACCTTGATTATCAGACTTTACA	c	c	
RD21_20206_4283_F<br>RD21_20206_4283_R	TGGAAACGAGTGCGATTAAG<br>TCTCAATCCGTTCCACCTCT	a	a	
RD21_20206_I2_F<br>RD21_20206_I2_R	GATGGAAAATGCGACAGTGCC<br>CAATCGAAACAACCTTCGCGTT	a	c	
RWA_29675_2498_F<br>RWA_29675_2498_R	TCCGGCAATCCTGATCTTAG<br>CATGGCTGGGAAAAATCACT	d	d	
sit_19807_6510_F<br>sit_19807_6510_R	TTCAACCACCTGTTTTGCAG<br>AATTCTGGGATTTGTTCAAGATT	a	a	**
sit_19807_I2_F<br>sit_19807_I2_R	GACACGTTGAGTGTGGTTCAA<br>TCCACCAAAACCTCACAACA	b	a	
SR1_1903_2688_F<br>SR1_1903_2688_R	AAGCCTGTTGGAACATGTTATG<br>TCAACAAGAATTGCAGCATTG	b	a	
SS_5198_1668_F<br>SS_5198_1668_R	GACCCAACTATGATAGCAGCAG<br>GCCTGATGAAACCCATCTTG	a	a	
SS_5198_2832_F<br>SS_5198_2832_R	CGCTTTTTGTAACACGGAAA<br>ATTATGGCCATCGGGACAG	a	a	
SSI2_11878_539_F<br>SSI2_11878_539_R	CTTCCACCCTTGGATCGTC<br>ATATTCTGCTCAGCCCAACC	c	c	
STP13_6824_1336_F<br>STP13_6824_1336_R	CTGAAGAGTACAGGCGCGTA<br>GTCGCATCGCTAAAGAAGTCA	c	c	
WRKY33_16847_1652_F<br>WRKY33_16847_1652_R	GGTTGCTTTGGACTTTCACC<br>GAACCCAATTGGAGCTTCTTT	b	a	

Table S3 (continued)

CGs markers	Primer	HRM results in parents		note
		S	F	
ZF_1325_432_F<br>ZF_1325_432_R	GCAACACGACTGTGCATCTT<br>ACGATTTCCGCCACCGTA	a	a	
ZF_30219_1665_F<br>ZF_30219_1665_R	CCCGTTGTCATCGGTTAAAT<br>CTGTGAATTCCGTTGAAACC	a	a	
ZF_30219_1910_F<br>ZF_30219_1910_R	TCGATCCGTTTCTTCTTCCA<br>ATGGGTTTGTATCGGAGGTG	c	a	
ZF_30683_646_F<br>ZF_30683_646_R	CAAGAGACGCCACTATGAAGG<br>ACCCTCCGACGAGGTGAC	c	c	
GAPDH_F<br>GAPDH_R	CCAGGAACCCAGAGGAGATACC<br>GGAGCGGATATGATGACCTTCTTG			**

* Four possible cases after PCR reaction and HRM analysis (a, b, c, d) see also Fig.S2
** Primer used for qRT-PCR

Table S4 Overview of mapped candidate genes, χ^2 test and *t*-test results

Candidate gene	hit	CGs marker*	χ^2(1:1)	mapped on parent_linkage group	*t*-test of the two genotypic groups+		
					Bottom	Ray Floret	Whole inflorescence
gh2-PS	contig11873	2_PS_11873_814	0.73	FP1_23	0.0052	0.7792	0.0528
ghACS	contig17688	ACS_17688_842	2.09	SP2_24	0.5025	0.2891	0.7043
ghAS	contig1926	AS_1926_I2	0.39	SP1_08	0.4974	0.6818	0.4380
ghCHI	contig22447	CHI_22447_421	0.08	FP2_23	0.1338	0.9597	0.0043
ghCHI	contig22447	CHI_22447_421	0.49	SP1_23	0.0506	0.3098	0.0090
ghCHS1	contig29143	CHS_29143_1582	0.04	SP1_17	0.0723	0.9820	0.0389
ghcutin	contig4918	cutin_4918_3081	3.06	SP2_02	0.0141	0.3986	0.2436
ghDND	contig29068	DND_29068_911	2.10	FP2_04	0.4132	0.7622	0.4412
ghDND	contig29068	DND_29068_911	0.62	SP2_04	0.2133	0.2612	0.0611
ghELP2	contig2398	ELP2_2398_1249	0.02	SP1_06	0.0174	0.3721	0.2071
ghERF	contig11093	ERF_11093_229	0.93	FP2_09	0.0075	0.2194	0.0583
ghET	contig14926	ET_14926_1910	0.00	FP1_16	0.3624	0.6401	0.2525
ghFDH	contig12215	FDH_12215_1158	0.00	SP1_02	0.0041	0.9751	0.0491
ghGSL5	contig30332	GSL5_30332_2672	2.79	FP1_20	0.4063	0.7067	0.2516
ghLOX	contig13375	LOX_13375_1313	0.10	SP1_12	0.1136	0.0120	0.0258
ghMPP3	contig15525	MPP3_15525_1055	0.82	SP2_01	0.7767	0.2504	0.7008
ghMYB	contig11297	MYB_11297_481	0.17	FP2_22	0.1389	0.6366	0.4696
ghP450	contig25584	P450_25584_2562	2.43	SP2_02	0.4991	0.0699	0.7834
ghPER21	contig12863	PER21_12863_774	3.28	FP2_05	0.9363	0.6885	0.2686
ghPER21	contig12863	PER21_12863_774	0.26	SP2_05	0.8169	0.0114	0.6455
ghPER62	contig31925	PER62_31925_540	1.71	FP2_18	0.9971	0.0016	0.4303
ghPG1	contig15001	PG1_15001_1052	0.81	FP2_21	0.6880	0.0002	0.7800
ghPG1	contig15001	PG1_15001_1052	0.00	SP1_21	0.2836	0.6503	0.1400
ghPG2	contig1158	PG2_1158_857	0.08	FP2_18	0.1045	0.0214	0.5778
ghPG7	contig35500	PG7_35500_1285	0.07	SP1_06	0.2745	0.4141	0.8631
ghPG9	contig25150	PG9_25150_888	2.32	SP2_02	0.0003	0.7148	0.0236

ghPG10	contig11147	PG10_11147_3429	0.01	FP2_06	0.3194	0.1243	0.4550
ghPG10	contig11147	PG10_11147_5246	0.04	SP1_06	0.0473	0.2727	0.2633
ghPGIP1	contig17627	PGIP1_17627_1064	0.33	SP2_22	0.3155	0.3981	0.0322
ghPGIP2	contig20957	PGIP2_20957_910	0.13	FP2_08	0.8248	0.9677	0.7393
ghRD21	contig20206	RD21_20206_4286	0.47	FP1_10	0.9234	0.4332	0.6737
ghRD21	contig20206	RD21_20206_I2	0.04	SP1_10	0.0679	0.2106	0.1072
ghsit	contig19807	sit_19807_6510	1.76	FP1_05	0.6503	0.0003	0.2911
ghsit	contig19807	sit_19807_I2	0.08	FP1_05	0.2024	0.0017	0.3657
ghsit	contig19807	sit_19807_6510	1.60	SP1_05	0.0134	0.0967	0.0835
ghSR1	contig1903	SR1_1903_2688	0.16	FP1_11	0.1517	0.2194	0.5784
ghSS	contig5198	SS_5198_1668	0.80	FP2_16	0.9438	0.8962	0.6704
ghSS	contig5198	SS_5198_1668	0.10	SP1_16	0.0009	0.3052	0.0119

* CG markers are named as: gene name_hit contig no._SNP position in the contig.
+ Significant results (p-value<0.05) are underlined

Table S5 QTLs found for bottom, whole inflorescence,and ray florets test in the parental genetic maps of two populations (after adding the new CG markers)

QTL	Flanking Markers	Linkage Group	MQM	
			LOD (GW)	% Expl.
RBQB1	WGC11243_647_S2F1a	SP1_02	5.19 (4.2)	7.3
RBQB2	WGC2476_271_S1	SP1_16	4.48 (4.2)	6.3
RBQB3	WGC18733_346_S2F	SP2_11	5.25 (4.0)	8.6
RBQB4	WGC16204_523_S2F1	FP1_01	6.87 (4.2)	10.4
RBQB5	WGC28102_213_S2F1	FP1_09	5.28 (4.2)	7.8
RBQB6	WGC18158_119_F1b	FP2_09	4.74 (4.1)	7.9
RBQRF1	WGC17798_117_S2F1	SP2_07	5.21 (4.0)	8.7
RBQRF2	***WGC22343_292_SFa, sit_19807_6510***	*FP1_05*	*6.81 (4.1)*	*8.9*
RBQRF3	WGC35370_146_S2F1	FP1_09	5.27 (4.1)	6.8
RBQRF4	WGC828_408_S2F	FP1_15	6.83 (4.1)	8.9
RBQRF5	WGC35264_283_S2F1, WGC6074_441_S2F	FP1_18	5.5 (4.1)	7.1
RBQRF6	WGC7520_3774_S1F2, WGC828_408_S2F	FP2_15	4.31 (4.1)	6.5
RBQRF7	WGC6074_441_S2F	FP2_18	4.09 (4.1)	5.9
RBQRF8	***PG1_15001_1052***	*FP2_21*	*6.42 (4.1)*	*10.7*
RBQWI1	WGC33030_228_S	SP1_11	4.74 (4.0)	7.2
RBQWI2	WGC1824_721_S1F1	SP1_23	5.46 (4.0)	8.4
RBQWI3	WGC18733_346_S2F	SP2_11	5.25 (4.0)	8.6
RBQWI4	WGC22447_285_Fa	FP1_23	6.96 (4.2)	11.4
RBQWI5	WGC5962_1153_F	FP2_17	5.34 (4.1)	8
RBQWI6	WGC1084_721_F	FP2_23	7.25 (4.1)	11
RBQB7*	***WGC19218_398_S1F1***	*SP1_06*	*5.33 (4.2)*	*7.6*
RBQB8*	***PG9_25150_888***	*SP2_02*	*4.0 (4.0)*	*6.1*

Note: Name of QTLs are RBQ (as Resistance Botrytis QTL) followed by the initials of disease tests used: B=Bottom; RF=Ray Floret; WI=Whole Inflorescence test. LG indicates linkage group and the LG number in the two populations ; Null-alleles are marked with a letter 'a' or 'b' in the end; GW indicates genome wide significant threshold level P<0.05; %Expl. is the percentage of total variance explained by the QTL. QTLs with * indication are the two new QTLs.

Table S6 The expression levels of three CGs on ray florets of gerbera

CGs	Parents	00hpi	06hpi	12hpi	24hpi	24hpi_mock
ghsit	SP1	0.97±0.22b	0.73±0.21b	1.32±0.36b	2.8±1.64**a**	0.52±0.04b
	SP2	1.15±0.68c	2.17±0.85b	1.88±0.57bc	4.03±1.68**a**	0.97±0.23c
	FP1	1.02±0.24b	2.51±0.32b	9.64±4.12**a**	-	1.42±0.2b
	FP2	1.02±0.22c	4.92±2.06b	17.44±5.63**a**	2.46±1.14c	0.93±0.22c
ghPG1	SP1	1.63±0.5c	3.98±2.1b	13.85±0.95**a**	4.3±0.22b	1.96±0.78bc
	SP2	1.12±0.49b	1.92±1.41b	2.19±2.31b	5.6±2.69**a**	3.16±3.22b
	FP1	-	-	-	-	-
	FP2	1.24±0.51c	4.61±4.23b	6.24±7.56**a**	3.12±1.61b	1.31±0.52c
ghCHI	SP1	0.97±0.26c	0.75±0.31bc	2.39±0.78**a**	1.48±0.34b	1.04±0.25bc
	SP2	1.3±0.59b	2.61±1.75b	2.59±1.28b	4.69±2.33**a**	1.38±0.63b
	FP1	-	-	-	-	-
	FP2	1.54±1.3b	1.68±1.13b	11.89±6.3**a**	1.49±0.83b	0.89±0.4b

Note: The expression levels of different timepoints were performed by pairwise comparisons, and the letters a, b, c, etc indicate the LSD test performance ($P<0.05$).

Table S7 The number of ray florets used for Botrytis disease test.

		Treatments			
		TRV2::GUS	*TRV2::ghPG1*	*TRV2::ghsit*	*TRV2::ghPG1+ghsit*
parents	SP1	27 (5)*	-	-	52 (10)
	SP2	22 (5)	21 (4)	20 (4)	84 (14)
	FP1	32 (5)	34 (6)	34 (6)	57 (10)

* the number of ray florets collected for disease test; the number in brackets are indicating from how many
